# RNA sequencing reveals molecular mechanisms of endometriosis lesion development in mice

**DOI:** 10.1242/dmm.050566

**Published:** 2024-10-23

**Authors:** Kavita Panir, John E. Schjenken, James Breen, Hon Yeung Chan, Erin Greaves, Sarah A. Robertson, M. Louise Hull

**Affiliations:** ^1^ Robinson Research Institute and School of Biomedicine, The University of Adelaide, Adelaide, SA 5006, Australia; ^2^ Adelaide Medical School, Faculty of Health and Medical Sciences, University of Adelaide, Adelaide, SA 5000, Australia; ^3^ School of Environmental and Life Sciences, College of Engineering, Science and Environment, The University of Newcastle, Callaghan, NSW 2308, Australia; ^4^ Infertility and Reproduction Research Program, Hunter Medical Research Institute, New Lambton Heights, NSW 2305, Australia; ^5^ South Australian Genomics Centre (SAGC), South Australian Health and Medical Research Institute (SAHMRI), Adelaide, SA 5000, Australia; ^6^ Computational and Systems Biology Program, Precision Medicine Theme, South Australian Health and Medical Research Institute (SAHMRI), Adelaide, SA 5000, Australia; ^7^ Centre for Early Life, Division of Biomedical Sciences, Warwick Medical School, University of Warwick, Coventry CV4 7AL, UK; ^8^ Department of Obstetrics and Gynaecology, Women's and Children's Hospital, Adelaide, SA 5006, Australia

**Keywords:** Endometriosis, Mouse model, RNA Sequencing, Immune regulation

## Abstract

Understanding of molecular mechanisms contributing to the pathophysiology of endometriosis, and upstream drivers of lesion formation, remains limited. Using a C57Bl/6 mouse model in which decidualized endometrial tissue is injected subcutaneously in the abdomen of recipient mice, we generated a comprehensive profile of gene expression in decidualized endometrial tissue (*n*=4), and in endometriosis-like lesions at Day 7 (*n*=4) and Day 14 (*n*=4) of formation. High-throughput mRNA sequencing allowed identification of genes and pathways involved in the initiation and progression of endometriosis-like lesions. We observed distinct patterns of gene expression with substantial differences between the lesions and the decidualized endometrium that remained stable across the two lesion timepoints, and showed similarity to transcriptional changes implicated in human endometriosis lesion formation. Pathway enrichment analysis revealed several immune and inflammatory response-associated canonical pathways, multiple potential upstream regulators, and involvement of genes not previously implicated in endometriosis pathogenesis, including *IRF2BP2* and *ZBTB10*, suggesting novel roles in disease progression. Collectively, the provided data will be a useful resource to inform research on the molecular mechanisms contributing to endometriosis-like lesion development in this mouse model.

## Teaser

**Summary:** This study utilizes high-throughput mRNA sequencing to identify genes and pathways involved in the initiation and progression of endometriosis-like lesions in a mouse model of endometriosis.

## INTRODUCTION

Endometriosis is a common condition that affects ∼11% of women of reproductive age (Rowlands et al., 2022). It is characterized by persistence of endometrium-like tissue at sites outside the uterine cavity, typically on the ovaries, fallopian tubes or other organs in the pelvic cavity (Saunders and Horne, 2021). A range of debilitating symptoms – including chronic pain, heavy menstrual bleeding and infertility – are typical (Zondervan et al., 2018) but not unique to endometriosis (Giudice, 2010). Despite improvements in ultrasound and magnetic resonance imaging, a conclusive diagnosis can take 6-10 years from the onset of symptoms (Nisenblat et al., 2016) and often involves invasive laparoscopic surgery. Effective treatment interventions have not been identified, and optimal treatment pathways remain under debate.

To design improved diagnostic and therapeutic approaches, better understanding of the disease pathogenesis is required (Duffy et al., 2021). Because there are insurmountable challenges associated with investigating disease etiology in humans, preclinical animal models are essential. Mice and other animals have been utilized to model different elements of the pathophysiological process, and to identify molecular and cellular pathways potentially involved in endometrial tissue attachment, proliferation and persistence in ectopic sites. These models enable formation of endometriosis-like lesions to be studied under controlled genetic and environmental conditions, generating important insight relevant to identifying targets for therapeutic interventions. A range of models utilize different strategies comprising human or mouse donor tissue delivered to peritoneal cavity or subcutaneous sites to recapitulate various elements of lesion formation and disease progression (reviewed in Bruner-Tran et al., 2012, 2018; Burns et al., 2021; Grümmer, 2006).

Immune system dysfunction is strongly implicated in the pathogenesis of endometriosis. Abnormalities in the levels and activity of several immune cells and cytokines have been identified in patients (Kralickova et al., 2018; Riccio et al., 2018; Zhang et al., 2018), but whether these are a cause or consequence of lesion development remains unclear. The initial steps in endometriotic lesion development involve endometrial-like tissue fragments presumed to arise from retrograde menstruation evading immune destruction. Impaired immune surveillance and aberrant cytokine expression likely contribute to the ability of ectopic endometrial cells to survive, proliferate and thrive in the peritoneal cavity (Aznaurova et al., 2014; Benagiano et al., 2014; Bouquet De Jolinière et al., 2014). Subsequently, immune cells appear to facilitate ectopic endometrial cell invasion of the mesothelial lining, cell proliferation and neovascularization of the tissue (Hull et al., 2003).

In particular, macrophages are implicated as a major driving force in the initiation and persistence of disease (Capobianco and Rovere-Querini, 2013). In part, this may be because macrophages secrete high concentrations of the prostaglandins F2α and E2 implicated in estrogen production required to sustain endometriotic lesion survival (Ferrero et al., 2014). Consistent with this, mouse models indicate dynamic roles for macrophages in lesion establishment and remodeling (Greaves et al., 2014; Haney et al., 1981; Hull et al., 2012; Koninckx et al., 2012; Kyama et al., 2003; van Furth and Cohn, 1968). Notably, studies using decidualized endometrium to generate homologous endometriosis-like lesions in mice have been useful in evaluating macrophage actions. This approach reveals a rapid infiltration of macrophages within the first few days of lesion initiation (Lin et al., 2006), but a shift in macrophage phenotype from a pro-inflammatory to a tissue-healing phenotype subsequently occurs (Johan et al., 2019). Reciprocal transfers between mice with GFP-labeled macrophages and wild-type mice shows that both donor and recipient macrophages are associated with endometriotic-like lesions (Greaves et al., 2014). Mouse studies have shown that neovascularization is critical to support lesion growth (Hull et al., 2003), and a role for macrophages in mediating lesion neovascularization, by virtue of their potent supply of vascular endothelial growth factor (VEGF), has been identified (Bacci et al., 2009; Capobianco and Rovere-Querini, 2013).

To expand understanding of the relevance and constraints of endometriosis-like lesions in mice, delineation of the transcriptomic profile accompanying lesion development is required. Sequencing data will enable identification of the molecular similarities and differences between lesions in mouse models and human disease and provide valuable insights into the mechanisms underlying lesion development and progression. In this study, we present RNA-sequencing (RNA-seq) analysis of mouse endometrial tissue representing the outset of lesion development, and endometriosis-like lesions at Day (D)7 and D14 after lesion induction. This information will advance understanding of the disease pathophysiology recapitulated in the mouse model, provide a resource to help identify new candidate therapeutic targets and biomarkers, and facilitate translation of findings from mouse studies to human patients.

## RESULTS

### Endometriosis-like lesions reduce in weight and size over time

**Fig. 1.**
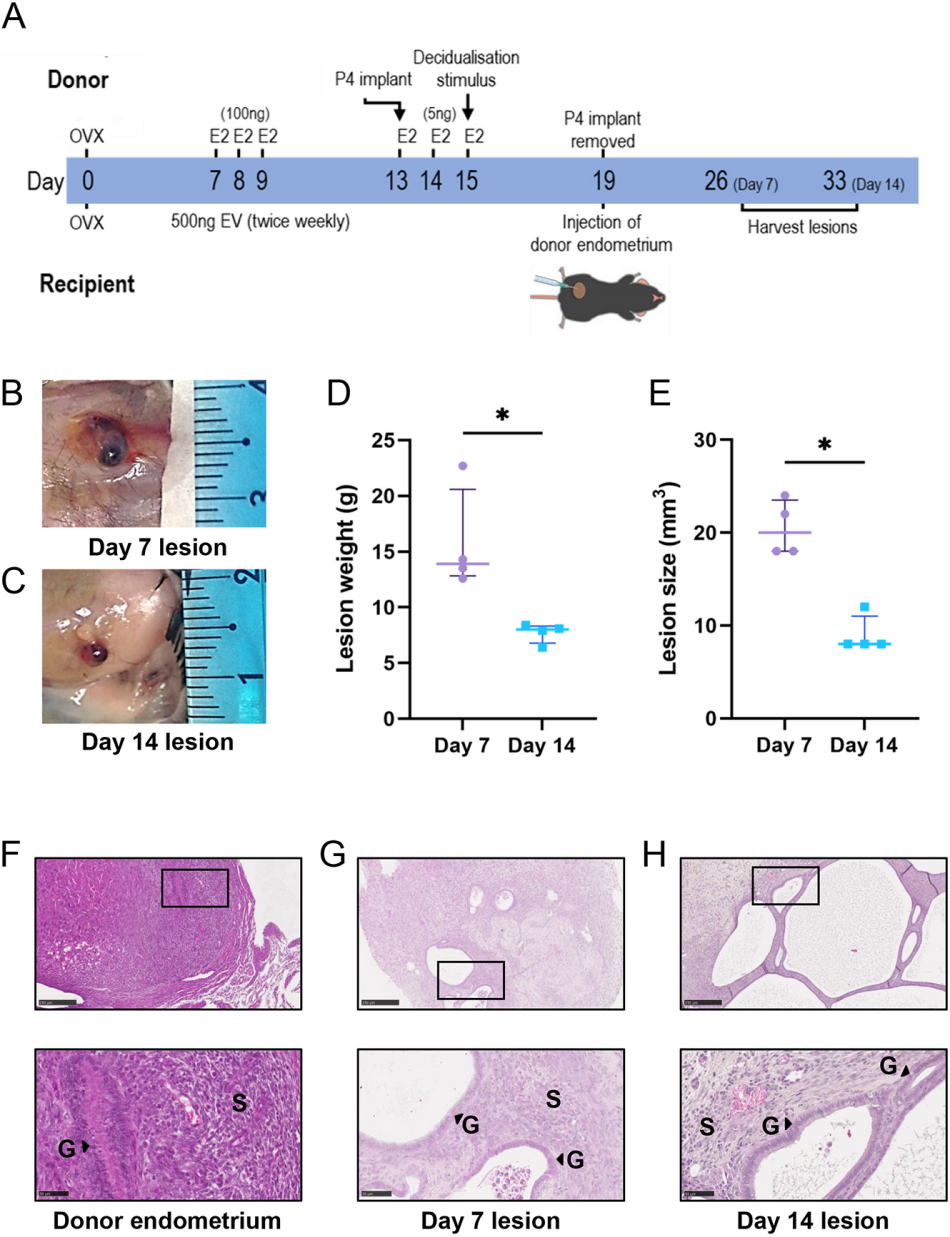
**Development of endometriosis lesions in mice.** (A) A modified version of a menstrual mouse model of endometriosis was used to establish subcutaneous endometriosis-like lesions in recipient mice. E2, beta-estradiol; EV, estradiol valerate; OVX, ovariectomy surgery; P4, progesterone. (B,C) Representative images of lesions at Day (D)7 (B) and D14 (C). (D,E) Lesions were excised, weighed (D) and measured (E). (F-H) Representative photomicrographs of Hematoxylin and Eosin (H&E)-stained sections from donor decidualized endometrium (F), D7 lesions (G) and D14 lesions (H). Top row: 10× magnification; bottom row: 40× magnification. Scale bars: 50 μm. G, glands; S, stroma. Data are presented as median±interquartile range, with each symbol representative of a single lesion in one mouse used for RNA sequencing (RNA-seq). Statistical analysis was performed using the Mann–Whitney test (*P<0.05).

We modified the well-established model of experimental endometriosis developed by Greaves et al. (2014) utilizing ‘menses-like’ donor decidualized endometrial tissue from hormone-treated donor C57Bl/6 mice. After excision from donor mice, 40 mg (±2 mg) fragmented decidualized endometrial tissue was implanted subcutaneously in the abdomen (lower-right quadrant) of ovariectomized recipient C57Bl/6 mice (Fig. 1A). Ovariectomy of recipient mice with exogenous E2 supplementation allowed for synchronization of a uniform hormonal status across the entire animal cohort, thus reducing variability associated with the estrus cycle. Previous work has shown that this approach overcomes the limitation of intraperitoneal inoculation, wherein endometrial tissue fragments often fail to successfully attach within the peritoneal cavity, resulting in non-vascularized, necrotic tissue devoid of proliferating cells (Burns et al., 2012), and a low lesion recovery rate (Grümmer et al., 2001). In the clinical setting, subcutaneous endometriosis, or ‘scar endometriosis’, can arise after disruption and dissemination of eutopic endometrium (e.g. following caesarean or laparoscopic surgery) or from pre-existing ectopic endometrial tissue transferred to incision sites during surgical resection, where it incorporates into the abdominal wall wound (Denton et al., 1990; Gaunt et al., 2004; Hull et al., 2006; Khammash et al., 2003; Liang et al., 1998). Subcutaneous inoculation of endometrial fragments in mice is considered a reasonable model of abdominal wall endometriosis, the incidence of which is rising in tandem with the increasing rate of caesarean sections and laparotomies (Carsote et al., 2020). Moreover, this approach allows encapsulation of tissue fragments between the skin and peritoneal lining, ensuring a lesion recovery rate of 63-100% (Hull et al., 2012) and facilitating accurate assessment of lesion size.

After lesion development, endometriosis-like lesions (*n*=4 at D7 and *n*=4 at D14; representative images shown in Fig. 1B,C, respectively) were carefully dissected, weighed and measured to determine lesion width, length and height prior to processing for RNA-seq. Median lesion weight was 13.9 mg at D7 and 8.0 mg at D14, a significant reduction across the time course (Fig. 1D). Likewise, lesions harvested at D7 were significantly larger than D14 lesions (20.0 mm^3^ versus 8.0 mm^3^) (Fig. 1E). Representative histochemically stained sections of decidualized endometrium harvested from donor mice (Fig. 1F) show the presence of decidualized stromal cells and luminal epithelium. Sections of D7 (Fig. 1G) and D14 (Fig. 1H) endometriosis-like lesions show the presence of glandular and stromal endometrial cells, consistent with lesion structures observed in human disease (Bulun, 2019).

### RNA-seq analysis of lesion progression

**Fig. 2.**
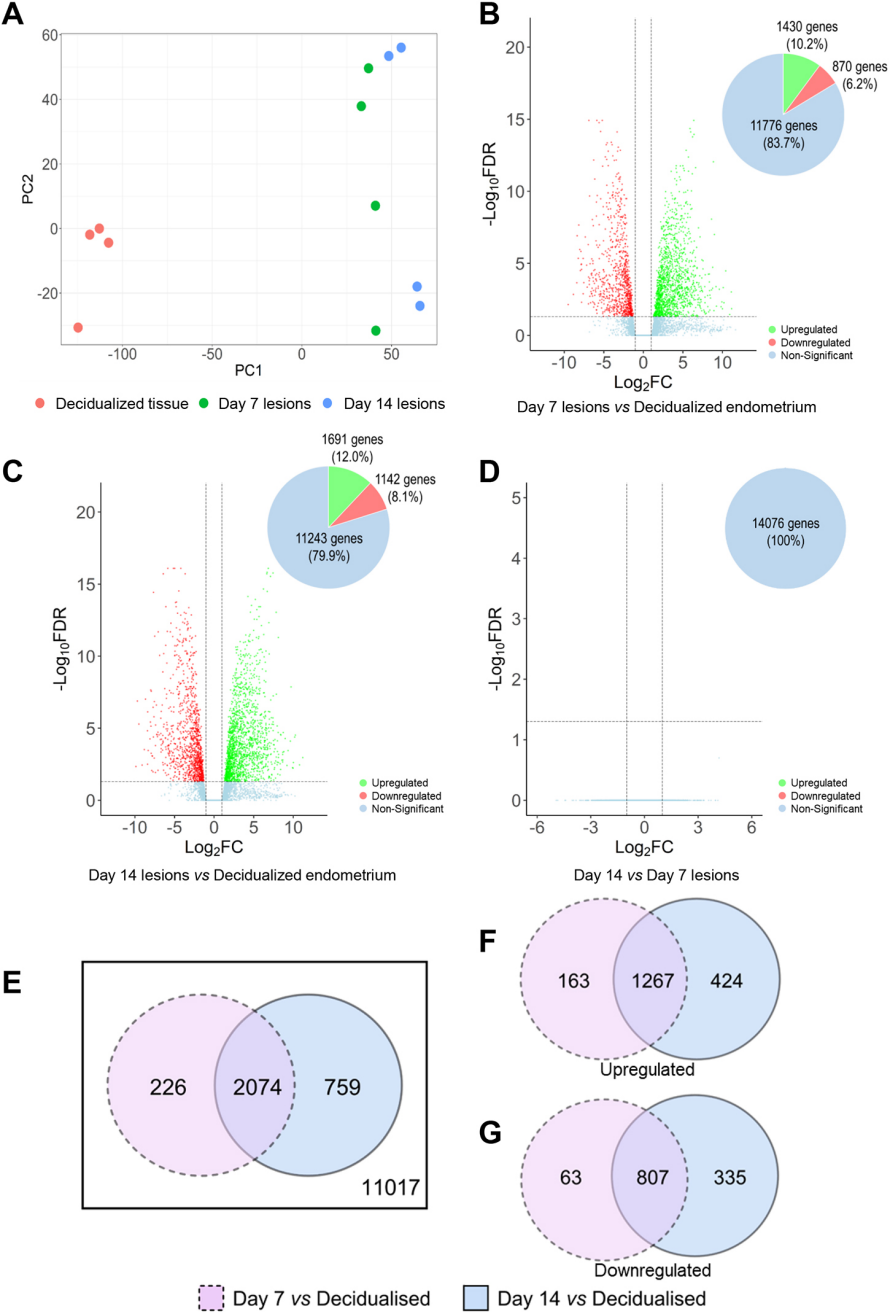
**Comparison of the gene expression profile in donor decidualized endometrium, and D7 and D14 endometriosis-like lesions.** Differentially expressed genes (DEGs) were identified using limma/voom and edgeR with a cut-off of false discovery rate-adjusted *P*-value (FDR)≤0.05 and log_2_fold change (log_2_FC)≥+1 or ≤−1. (A) Principal component (PC) analysis of RNA-seq results visualizing the gene expression pattern of individual samples used in this analysis. (B-D) Volcano plots comparing log_10_FC and −log_10_(FDR) among detected genes between D7 lesions versus decidualized endometrium (B), D14 lesions versus decidualized endometrium (C), and D14 lesions versus D7 lesions (D). (E) Venn diagram displays the distribution and overlap of DEGs (both upregulated and downregulated) between D7 lesion versus decidualized endometrium and D14 lesion versus decidualized endometrium. (F,G) Additional Venn diagrams were generated to determine the number of upregulated (F) and downregulated (G) DEGs during lesion development compared to decidualized endometrial tissue. A complete list of DEGs can be found in Tables S1-S3.

To assess the molecular changes associated with lesion development, RNA-seq was performed on donor decidualized endometrial tissue, D7 lesions and D14 lesions. Following alignment to the mouse reference genome and filtering to remove genes exhibiting low expression, a total of 14,076 genes were identified from the RNA-seq analysis. Average gene expression was obtained (*n*=4 samples in each of three groups of donor endometrium, D7 and D14 lesions), and the proportion of differentially expressed genes (DEGs) between groups was determined using a fold change in expression threshold of ≥+2 or ≤−2, with a false discovery rate-adjusted *P*-value (FDR)≤0.05 as the cut-off. Principal component analysis (PCA) using normalized RNA-seq data showed clustering of donor decidualized endometrial tissue with distinct separation from both D7 and D14 lesions (Fig. 2A; additional PCA in Fig. S1). A comparison of D7 endometriosis-like lesions to decidualized endometrium showed upregulation in 10.2% of detected genes and downregulation in 6.2% of detected genes (Fig. 2B). Between D14 lesions and decidualized endometrium, a total of 12% of detected genes were upregulated and 8.1% of detected genes were downregulated (Fig. 2C). In contrast, between D14 and D7 lesions, none of the detected genes were differentially expressed (Fig. 2D).

Comparisons of DEGs identified from D7 versus decidualized endometrium and D14 versus decidualized endometrium identified consistent dysregulation in 14.7% (2074) of all detected genes, with an additional 1.6% (226) DEGs unique to D7 versus decidualized endometrium and 5.4% (759) DEGs unique to D14 versus decidualized endometrium (Fig. 2E). A further division of DEGs between D7 versus decidualized endometrium and D14 versus decidualized endometrium into upregulated (1854) genes and downregulated (1205) genes was undertaken (Fig. 2F,G, respectively). Proportionally, 68.3% (1267 genes) of upregulated genes were consistently upregulated in lesions at both timepoints, while 67.0% (807 genes) of the downregulated genes were consistently downregulated in both D7 and D14 lesions compared to decidualized endometrium.

The genes with the largest fold change in expression between the endometriosis-like lesions and decidualized endometrium were identified (Table 1; for complete list of DEGs see Tables S1-S3). When compared with decidualized endometrium, lesions at both D7 and D14 had C1q tumor necrosis factor-related protein 3 (*C1qtnf3*; involved in gluconeogenesis and cell communication), dermatopontin (*Dpt*; involved in cell–matrix interactions and matrix assembly), superoxide dismutase 3 (*Sod3*; involved in response to hypoxia) and T-box transcription factor 15 (*Tbx15*; involved in regulation of developmental processes) among the top 10 upregulated DEGs. Expression of beta-carotene oxygenase 1 (*Bco1*; involved in beta-carotene metabolic process), prolactin family 3, subfamily c, member 1 (*Prl3c1*; involved in hormone activity, regulation of proliferation and decidual cell differentiation), prostate stem cell antigen (*Psca*; involved in regulation of neurotransmission) and tachykinin 2 (*Tac2*; involved in the regulation of blood pressure) were amongst the top 10 downregulated DEGs in both D7 and D14 endometrial lesions compared to decidualized endometrial tissue.

**Table 1.**
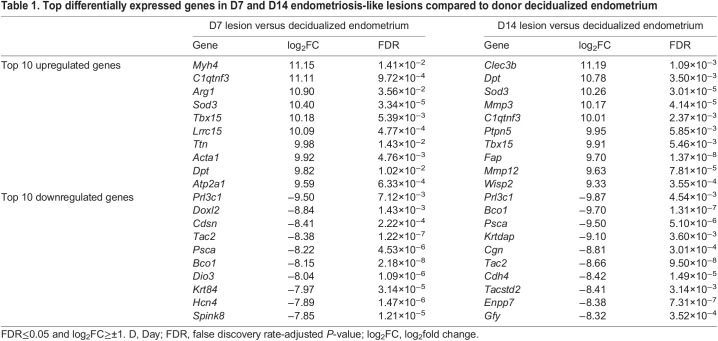
Top differentially expressed genes in D7 and D14 endometriosis-like lesions compared to donor decidualized endometrium

To determine the relevance of this model in emulating human disease, mouse DEGs common to both D7 and D14 versus endometrium [log_2_fold change (log_2_FC)≥±2, FDR≤0.05] were converted to human orthologs. This conversion resulted in the identification of 927 upregulated and 526 downregulated unique genes. From this list, a subset of immune-associated genes was compared to genes expressed in human endometriosis lesion datasets, utilizing the EndometDB database (Gabriel et al., 2020). Heatmaps were generated to visualize the expression patterns of these orthologous genes in patient endometrium and peritoneal lesions (Fig. S2), allowing comparison of the expression profiles between mouse and human tissues to interrogate the translational relevance of the model. The expression pattern of upregulated genes (Fig. S2A) demonstrates a considerable degree of conservation between human and mouse tissues. Notably, *DPT* exhibited significantly elevated expression in peritoneal endometriosis lesions and was mirrored by its homolog *Dpt* ranking prominently among the top upregulated genes in the mouse model. Several other genes, including complement genes *C1QA*, *C1QB* and *C1QC*, immune regulatory molecules *CD40* and cytokine *IL34*, showed similar patterns, with elevated expression in lesions compared to eutopic endometrium in human as well as mouse tissues. Among more than 50 genes evaluated, only one gene demonstrated an opposing pattern in mouse. *Arhgef19*, a gene implicated in signaling pathways related to cell migration and cytoskeleton organization, was elevated in mouse endometriosis-like lesions compared to decidual tissue, while *ARHGEF19* is downregulated in peritoneal endometriosis lesions in human (Fig. S2A). This underscores that the upregulated genes demonstrate a remarkable degree of conservation between mouse and human disease models.

Greater variation was observed among the downregulated homologs (Fig. S2B). Specifically, the mouse model faithfully recapitulated the expression profiles of asparaginase like 1 (*ASRGL1*; involved in regulating asparagine metabolism and potentially affecting cellular proliferation and migration), claudin 10 (*CLDN10*; roles in regulating cellular permeability) and deltex E3 ubiquitin ligase 1 (*DTX1*; involved in modulating the Notch signaling pathway and potentially influencing cell fate determination). Conversely, genes including dystrobrevin alpha (*DTNA*; implicated in maintaining muscle structure and function), cadherin 3 (*CDH3*; involved in mediating cell–cell adhesion and tissue morphogenesis), claudin 1 (*CLDN1*; roles in regulating epithelial barrier function) and optineurin (*OPTN*; involved in vesicle trafficking, NF-κB signaling and autophagy) exhibited contrasting expression patterns and were upregulated in human lesions compared to the downregulation observed in the mouse model. Thus, although many downregulated genes exhibit between-species conservation, there was not complete identity between the mouse and human lesion expression patterns.

**Fig. 3.**
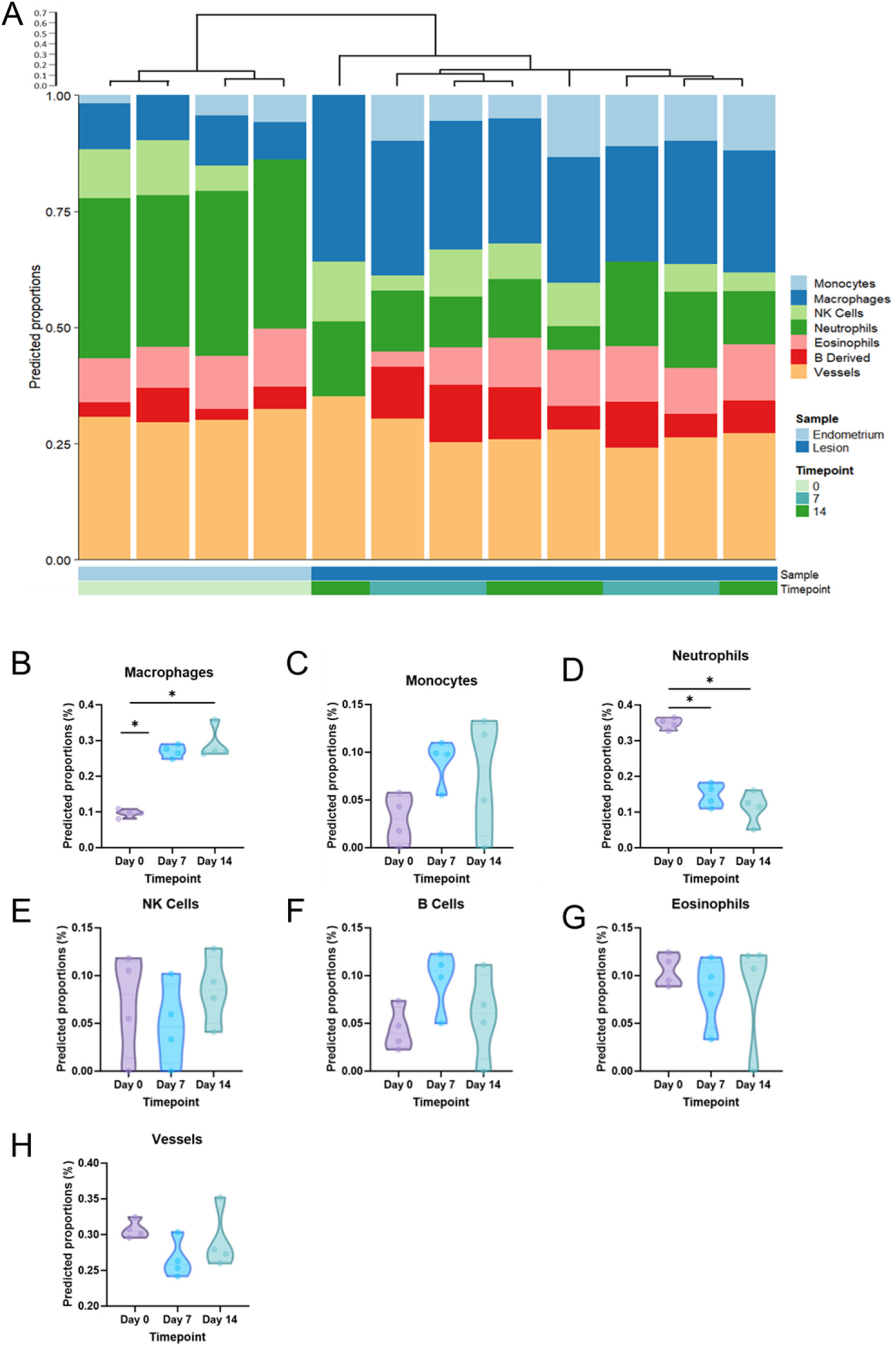
**Cellular deconvolution of RNA-seq data.** (A) Cellular deconvolution with Euclidean clustering was performed for each of the 12 tissue samples. (B-H) Predicted proportions of macrophages (B), monocytes (C), neutrophils (D), natural killer (NK) cells (E), B cells (F), eosinophils (G) and vessels (H) were compared across the three timepoints (D0, decidualized endometrium; D7, Day 7 lesions; D14, Day 14 lesions). Effect of tissue status was analyzed by Mann–Whitney test (*P<0.05).

Next, a cellular deconvolution algorithm was applied to the dataset to estimate cell-type contributions to the bulk RNA-seq data (Fig. 3A). Macrophages (Fig. 3B) emerged as the predominant predicted immune cell population in both D7 and D14 lesions, showing a significant increase compared to D0 (decidualized endometrium). Similarly, monocytes (Fig. 3C) exhibited a predicted upregulation in lesions, although not reaching statistical significance compared to decidualized endometrium. In contrast, neutrophils (Fig. 3D) were predicted to be significantly fewer in lesions than in decidualized endometrium. No significant differences were observed in the predicted populations of natural killer (NK) cells, B cells, eosinophils or vessels (Fig. 3E-H).

### Canonical pathways regulated during endometriosis-like lesion progression

**Fig. 4.**
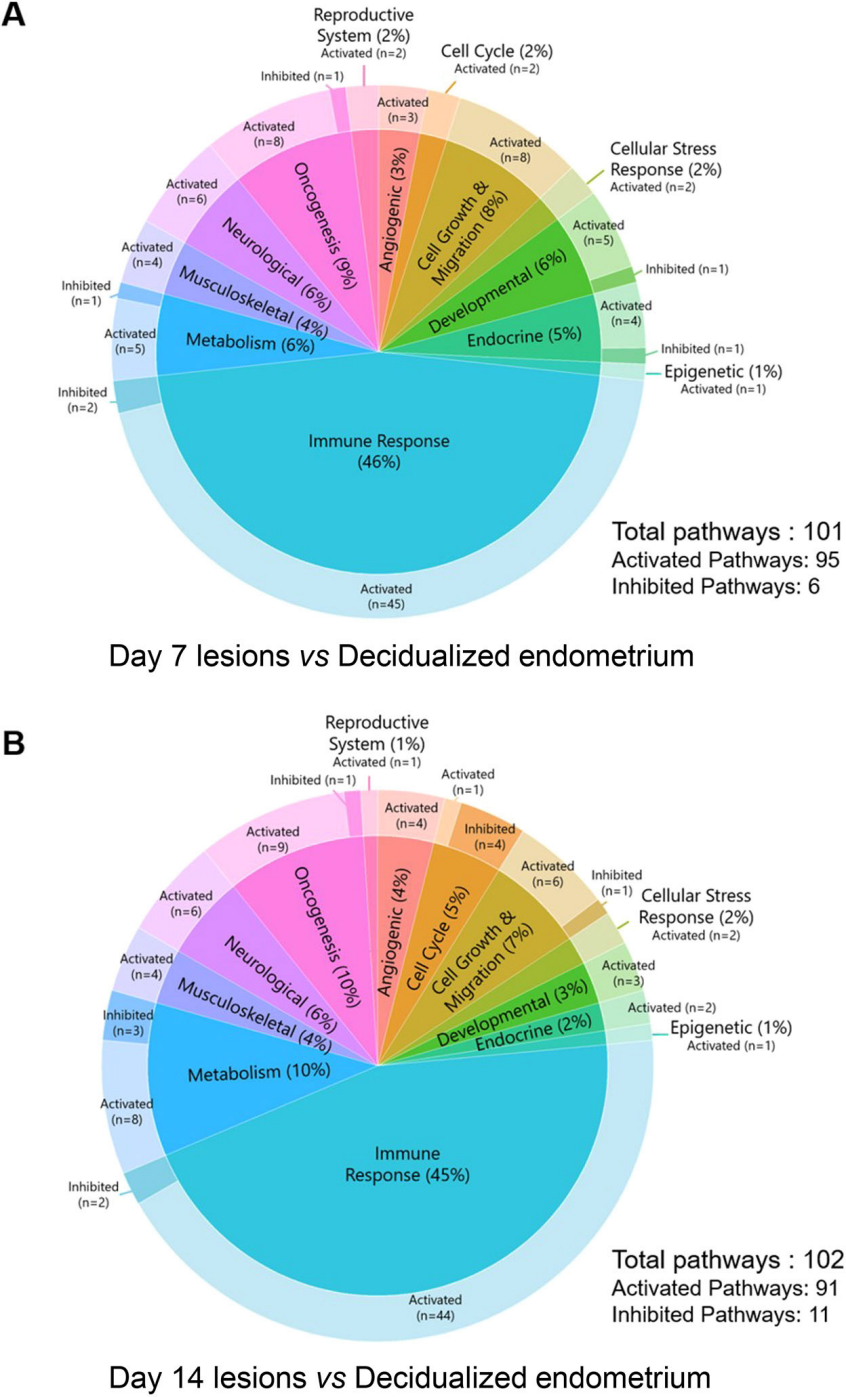
**Classification of canonical pathways identified by Ingenuity Pathway Analysis (IPA).** (A,B) Canonical pathways identified in D7 endometriosis-like lesions versus decidualized endometrium (A), and D14 endometriosis-like lesions versus decidualized endometrium (B). The full list of canonical pathways is available in Tables S4 and S5. All pathways shown have a *Z*-activation score of ≥+2 or ≤−2, and corresponding *P*-value of ≤0.05.

To understand and characterize the functional relevance of the transcriptional changes during endometriosis-like lesion development, Ingenuity Pathway Analysis (IPA) was used to infer canonical pathways regulated by the differentially expressed genes (*Z*-activation score of ≥+2 or ≤−2, and corresponding *P*-value of ≤0.05). A total of 95 pathways were predicted to be upregulated, and six pathways were predicted to be inhibited, in D7 lesions compared to decidualized endometrial tissue (Fig. 4A; Table S4); while 91 pathways were predicted to be upregulated, and 11 pathways were predicted to be inhibited, in D14 lesions compared to decidualized endometrial tissue (Fig. 4B; Table S5). Most of the identified pathways were common between both comparisons, with a total of 72 identical pathways activated in both comparisons, while three pathways (‘Antioxidant Action of Vitamin C’, ‘Inhibition of Matrix Metalloproteases’ and ‘PD-1, PD-L1 cancer immunotherapy pathway’) were inhibited in both comparisons. A predominance of canonical pathways associated with immune responses was observed in both D7 versus decidualized endometrium and D14 versus decidualized endometrium (46% and 45%, respectively), followed by oncogenic pathways (9% and 10%, respectively), metabolic pathways (6% and 10%, respectively), cellular growth and migration pathways (8% and 7%, respectively), and neurological pathways (6% for both). Angiogenic, cell cycle, cellular stress response, developmental, endocrine, epigenetic, musculoskeletal and reproductive system pathways were also identified (Fig. 4A,B).

**Fig. 5.**
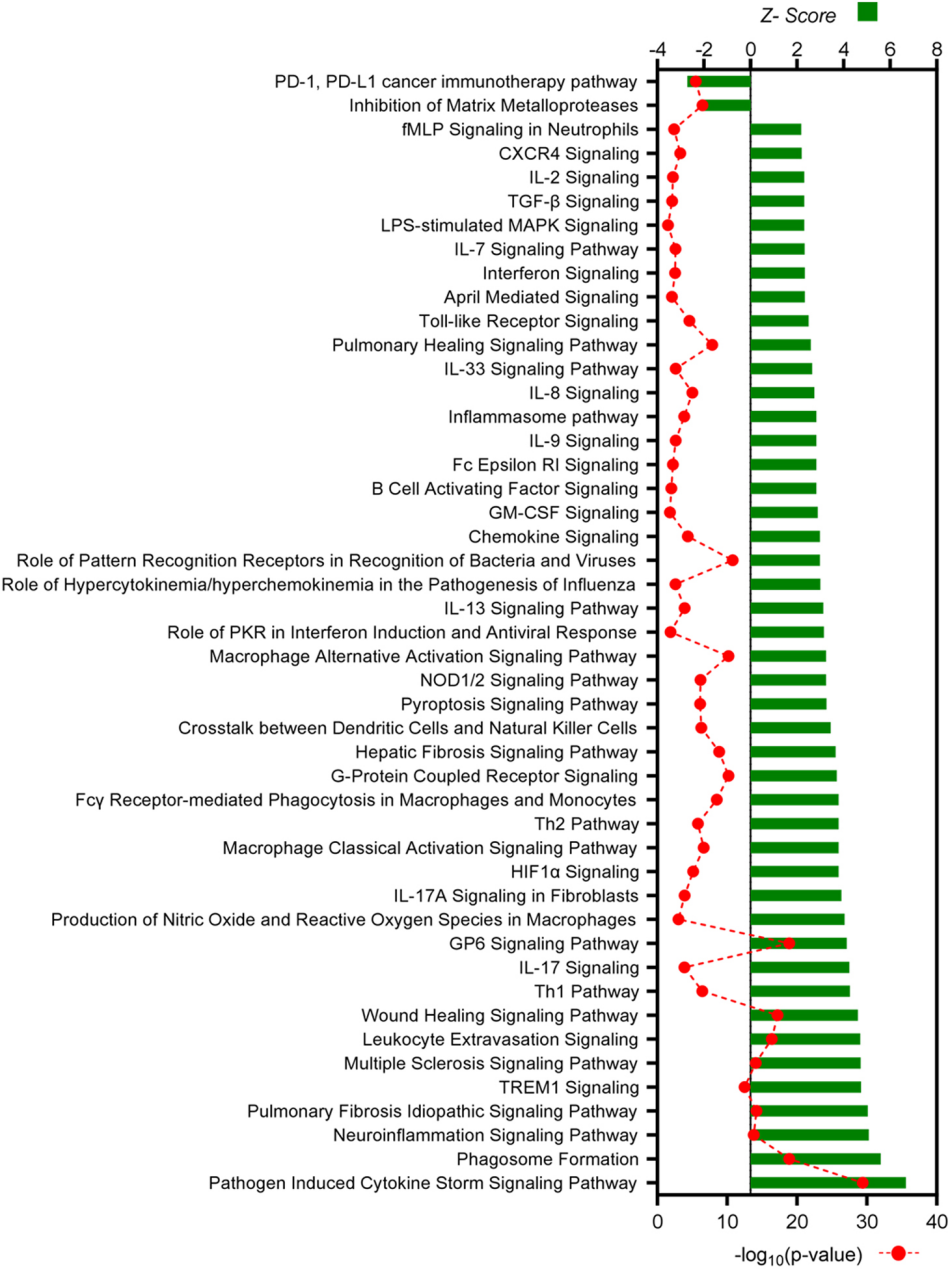
**All canonical pathways associated with immune responses in D7 endometriosis-like lesions versus decidualized endometrium identified by IPA.** Canonical pathways predicted to be associated with immunological response are listed, with *Z*-score shown on the top axis and −log_10_(*P*-value) on the bottom axis. All pathways shown have a *Z*-activation score of ≥+2 or ≤−2, and corresponding *P*-value of ≤0.05. The full list of canonical pathways is available in Table S4.

**Fig. 6.**
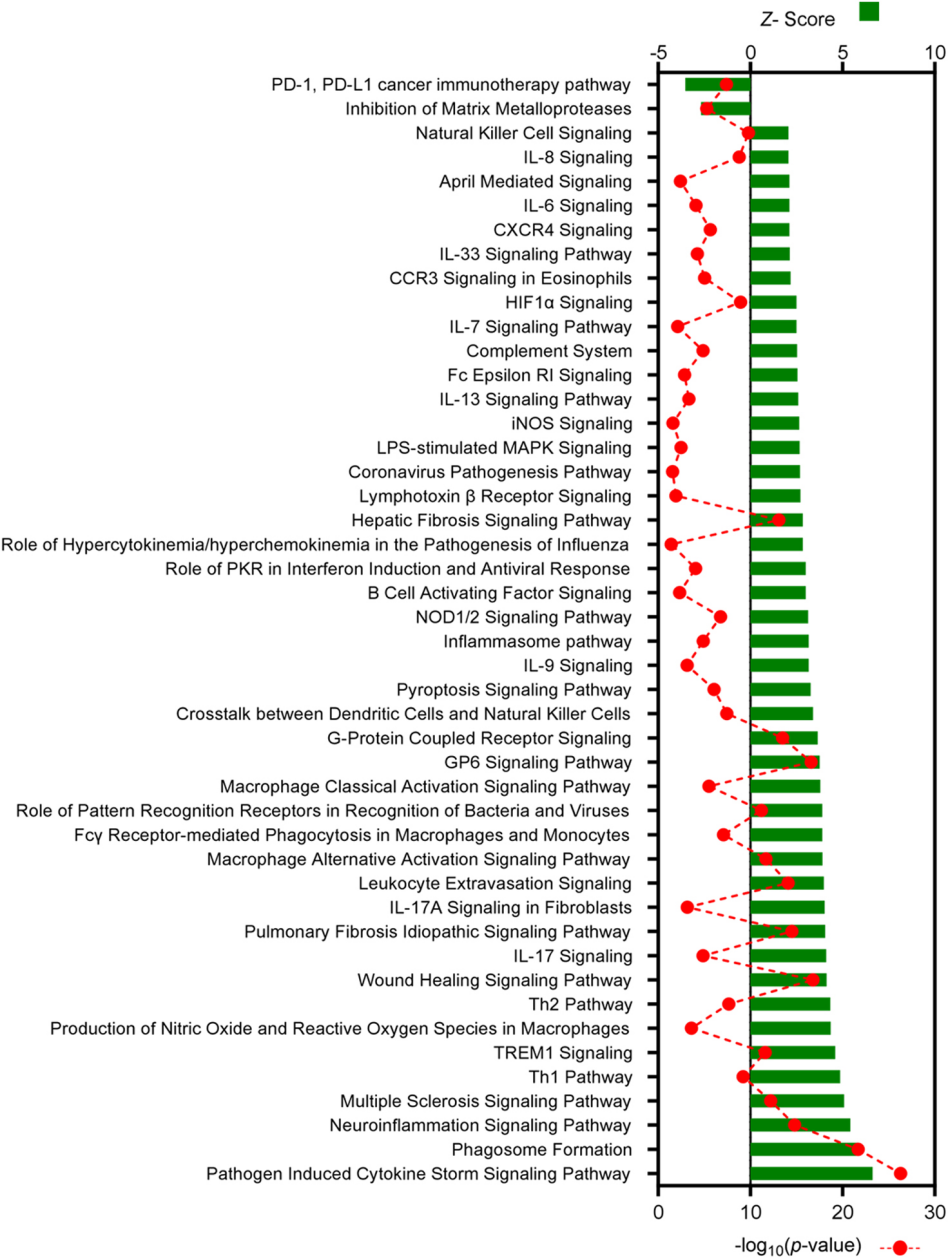
**All canonical pathways associated with immune responses in D14 endometriosis-like lesions versus decidualized endometrium identified by IPA.** Canonical pathways predicted to be associated with immunological response are listed, with *Z*-score shown on the top axis and −log_10_(*P-*value) on the bottom axis. All pathways shown have a *Z*-activation score of ≥+2 or ≤−2, and corresponding *P*-value of ≤0.05. The full list of canonical pathways is available in Table S5.

Pathways associated with immune responses comprised the largest proportion of identified canonical pathways and were evaluated in more detail (Figs 5 and 6). In both comparisons, two similar immunological pathways were identified as being inhibited: the ‘PD-1, PD-L1 cancer immunotherapy pathway’ and the ‘Inhibition of Matrix Metalloproteases’ pathway. In contrast, a total of 45 immune-associated pathways were predicted to be activated in endometriosis-like lesions at D7, and 44 pathways were predicted to be activated at D14, compared to decidualized endometrium. The two comparisons shared 37 immune-associated pathways, with ‘Pathogen Induced Cytokine Storm Signaling Pathway’, ‘Phagosome Formation’ and ‘Neuroinflammation Signaling Pathway’ being the top three in both analyses. Several additional canonical pathways identified in both datasets were strongly associated with monocyte and macrophage activity including ‘Fcγ Receptor-mediated Phagocytosis in Macrophages and Monocytes’, ‘Macrophage Alternative Activation Signaling Pathway’, ‘Macrophage Classical Activation Signaling Pathway’, ‘Leukocyte Extravasation Signaling’ and ‘Production of Nitric Oxide and Reactive Oxygen Species in Macrophages’. Among the eight pathways unique to D7 endometriosis-like lesions were ‘GM-CSF Signaling’, ‘IL-2 Signaling’, ‘Interferon Signaling’ and ‘TGF-β Signaling’, while the pathways ‘IL-6 Signaling’, ‘iNOS Signaling’ and ‘Natural Killer Cell Signaling’ are some of the seven pathways unique to D14 endometriosis-like lesions.

### Upstream molecules regulated during endometriosis-like lesion progression

**Fig. 7.**
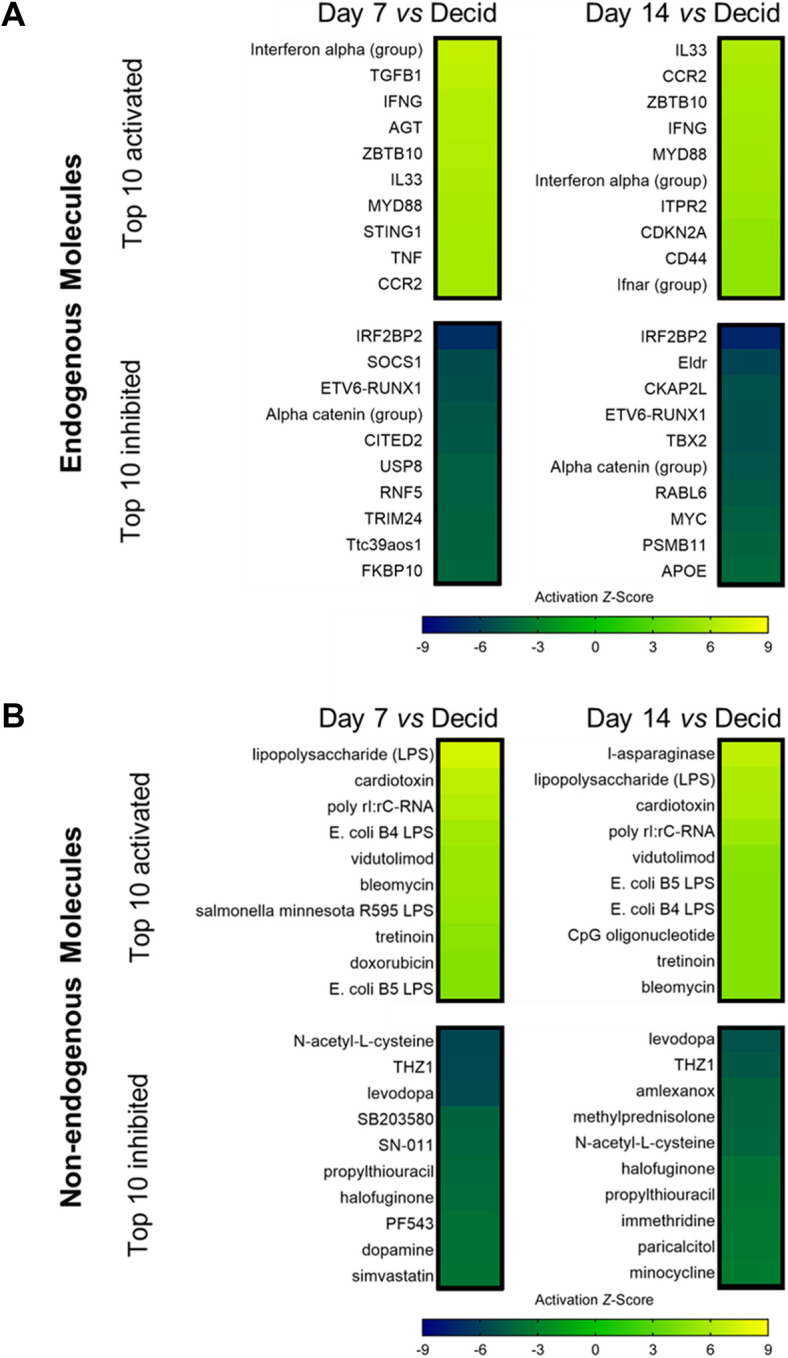
**Molecules predicted to be upstream regulators of differentially expressed genes in Day 7 and Day 14 endometriosis-like lesions versus decidualized endometrium identified by IPA.** (A) Heatmaps of the top 10 activated and top 10 inhibited endogenous upstream regulators are shown for D7 lesion versus decidualized endometrium and D14 lesion versus decidualized endometrium. (B) Heatmaps of the top 10 activated and top 10 inhibited non-endogenous upstream regulators are shown for D7 lesion versus decidualized endometrium and D14 lesion versus decidualized endometrium. All upstream regulators have a *Z*-activation score of ≥+2 or ≤−2, and corresponding *P*-value of ≤0.05. The full list of upstream regulators is available in Tables S6 and S7.

Upstream regulators implicated in contributing to endometriosis-like lesion progression were identified using IPA upstream regulator analysis, using criteria of *Z-*score of ≥+2 or ≤−2, and corresponding *P*-value of ≤0.05, and were classified into 28 molecular categories (Fig. S3). From this analysis, 921 molecules (62% activated) were predicted upstream drivers of the observed gene expression changes in D7 lesions versus decidualized endometrium (Table S6), and 806 molecules (64% activated) were predicted upstream drivers of the observed gene expression changes in D14 lesions versus decidualized endometrium (Table S7). Of the total 1134 upstream regulators identified, 591 (52%) were common across both comparisons, with the top 10 activated and top 10 inhibited endogenous and non-endogenous molecules shown in Fig. 7A and B, respectively. Additional heatmaps were generated to visualize these comparisons for the categories of transcription regulators, cytokines, microRNAs, receptors and growth factors (Fig. S4).

Activation of endogenous upstream regulators C-C motif chemokine receptor 2 (CCR2), interferon gamma (IFNG), interleukin 33 (IL-33), interferon alpha-group, MYD88 innate immune signal transduction adaptor (MYD88) and zinc finger and BTB domain-containing 10 (ZBTB10) was predicted for both D7 and D14 lesions compared to decidualized endometrium, while inhibition of Alpha catenin group, and transcription factors ETV6-RUNX1 and IRF2BP2, was predicted at both timepoints (Fig. 7A). Non-endogenous activated upstream regulators conserved between both timepoints included several compounds known to elicit a potent inflammatory response, such as lipopolysaccharides (LPS), cardiotoxin, bleomycin and poly rI:rC-RNA, while drugs such as N-acetyl-L-cysteine, halofuginone and levodopa were among the inhibiting upstream regulators (Fig. 7B).

From these lists of top predicted endogenous and non-endogenous upstream regulators, we identified many agents that have not yet been investigated or implicated in the pathogenesis of endometriosis. We achieved this via a PubMed search using the individual molecule identifiers listed in Fig. 7 coupled with the term ‘endometriosis’ (e.g. ‘IRF2BP2’ AND ‘endometriosis’), and the number of search results/publications was recorded (Fig. S5 for endogenous molecules and Fig. S6 for non-endogenous molecules). From the top endogenous upstream regulators identified from both D7 and D14 comparisons, there were 11 molecules with ≥5 search results returned, the highest of which was TNF, followed by CD44, MYC, TGFB1 and IL33 (Fig. S5). There were 12 molecules that returned no search results on PubMed, including the transcription regulators IRF2BP2, TBX2 and ZBTB10, suggesting that these endogenous molecules could have as yet unidentified roles in the establishment of endometriosis lesions. From the top 27 non-endogenous upstream regulators identified in both D7 and D14 comparisons, eight molecules returned ≥5 search results, including LPS, dopamine, doxorubicin, tretinoin and simvastatin (Fig. S6). A total of 14 molecules did not yield search results, including amlexanox, cardiotoxin, halofuginone, immethridine, paricalcitol and vidutolimod, suggesting potential utility as novel chemical interventions with possible therapeutic properties in endometriosis.

## DISCUSSION

In this study, we characterized by RNA-seq the transcriptomic changes that occur during the development of endometriosis-like lesions induced by subcutaneous placement of ‘menses-like’ decidualized endometrial tissue in mice. We found substantial differential expression of genes between the lesions and the decidualized endometrium from which they originate, many of which mimic pathways known to be involved in human disease (Mishra et al., 2020). In contrast, the absence of gene expression differences between D7 and D14 lesions suggests that, after significant changes during lesion development, the underlying transcriptional pathways remain relatively constant after the first week.

In concordance with published findings, this RNA-seq study demonstrates a central role for aberrant immune responses in endometriosis-like lesion development, as pathways associated with immune responses comprised the largest proportion of identified canonical pathways. Impaired immune surveillance, cytokine and leukocyte perturbations, and resistance of endometrial tissue to leukocyte-mediated clearance have all been implicated in the pathogenesis of endometriosis (Aznaurova et al., 2014; Benagiano et al., 2014; Bouquet De Jolinière et al., 2014). Local inflammation, associated with altered functional states in immune cells within the peritoneal cavity, is a paramount feature of endometriosis (Zondervan et al., 2018). Notably, we observed dysregulation of genes associated with both pro-inflammatory and pro-healing macrophage phenotypes during lesion development. Given the subcutaneous site of tissue placement, this did not depend on cells or factors specific to the peritoneal cavity, consistent with contemporary understanding that infiltrating monocytes are key players in both the progression and resolution of inflammatory responses regardless of tissue location (Cao et al., 2004; Jantsch et al., 2014). Our gene expression findings reflect the expected recruitment and differentiation of monocytes and subsequent heterogeneity of resident macrophage subpopulations as described previously for lesions established in both subcutaneous and peritoneal sites (Johan et al., 2019; Hogg et al., 2021; Panir et al., 2022; Henlon et al., 2024).

Furthermore, CD45^+^ (PTPRC^+^) single-cell sequencing of murine endometriosis-like lesions and peritoneal fluid (Henlon et al., 2024) reveals a recurrent wound healing cycle. Monocytes infiltrate, differentiate into pro-inflammatory-like macrophages and transition into pro-repair macrophages steadily over time. This mirrors findings from a subcutaneous xenograft mouse model (Johan et al., 2019), indicating the influence of the ectopic endometrial-like tissue microenvironment on immune cell differentiation dynamics. Additionally, a ‘protective’ monocyte-derived large peritoneal macrophage (LpM) population is identified in induced endometriosis models (Hogg et al., 2021). The absence of such LpMs in the subcutaneous model potentially reflects the pathophysiological conditions observed in women with endometriosis, where lesions establish and persist ectopically. Conversely, the peritoneal model may better mimic conditions in women without endometriosis, marked by lesion resolution. Therefore, we postulate that the identified DEGs are likely to be relevant to human disease, as tissue remodeling and survival mechanisms in subcutaneous tissue resemble those in the peritoneal cavity. Notably, we contend that recruited monocytes’ phenotype and function in endometrial tissue are dictated by the tissue environment rather than the external milieu, making the subcutaneous model relevant in evaluating mechanisms driving ectopic endometrial tissue survival and disease establishment.

Several pathways implicated by the DEGs in D7 tissues compared to decidualized tissue reflect hypoxic stress responses to disruption and displacement of endometrial tissue fragments transferred to subcutaneous sites. This reflects similar adjustments required of human endometrial fragments as they leave the uterus and take up residence in the peritoneal cavity. In its eutopic condition, endometrial tissue thrives in the well-vascularized and oxygenated microenvironment of the uterus. Under hypoxic conditions, cells activate a range of survival mechanisms, including angiogenesis, steroidogenesis, metabolic switching and epigenetic modulation, to prevent necrosis and death (Wu et al., 2019). Consistent with hypoxia driving the early phases of ectopic endometrial tissue survival, hypoxia induced factor-1α (*HIF1A*) gene expression is upregulated during tissue breakdown in a mouse model of menstruation (Chen et al., 2015b). In our dataset, we observed activation in the ‘HIF1α Signaling Pathway’ and ‘Production of Nitric Oxide and Reactive Oxygen Species in Macrophages’ pathway, accompanied by elevated expression of *Sod3* in lesions at both D7 and D14 timepoints. The superoxide dismutase (SOD) antioxidant enzymes catalyze conversion of superoxide radicals and reactive oxygen species (ROS) into hydrogen peroxide and oxygen, protecting tissues from damage caused by oxidative stress (Nguyen et al., 2020). Low levels of ROS are associated with angiogenesis in the endometrium, facilitating tissue regeneration during each reproductive cycle, but elevated ROS concentrations can impact fertility outcomes (Gupta et al., 2014). Endometriosis is associated with decreased SOD activity in peritoneal fluid compared to that of fertile women (Liu et al., 2001; Szczepańska et al., 2003), and reduced SOD expression in the uterus is linked with aberrant endometrial function due to elevated oxidative stress (Chandra et al., 2009; Gupta et al., 2014). Our finding of reduced *Sod3* expression in decidualized endometrium compared to lesions challenges the narrative linking decreased peritoneal SOD3 activity with endometriosis progression. Although clearly the significance to human disease is limited given the constraints of the model, this observation suggests that future studies should investigate both peritoneal and lesion SOD to achieve a more comprehensive understanding of the interplay between SOD and hypoxia.

We and others have previously reported a shift in the predominating immune status from pro-inflammatory to pro-reparative as endometriosis-like lesions become established and adapt to a hypoxic environment after subcutaneous placement in mice (Johan et al., 2019). In the initial phase of a wound healing response, macrophages typically secrete a range of pro-inflammatory cytokines – including IL-1, IL-6, IL-12 and TNF – to promote clearance of damaged tissues and progression to resolution (Cao et al., 2004). In women, phagocytosis of necrotic endometrial cells is associated with a decrease in the pro-inflammatory cytokines IL-1β and TNF, suggesting that the degree of inflammatory activation declines but never fully resolves over time (Capobianco and Rovere-Querini, 2013). This supports the concept that endometriotic lesions are comparable to wounds undergoing repeated tissue injury and repair (Ding et al., 2015; Zhang et al., 2016b). A shift from a pro-inflammatory milieu towards a pro-reparative environment in endometriosis lesions is accompanied by elevated expression of transforming growth factor β1 (TGF-β1) (Zhang et al., 2016a) and α-smooth muscle actin (α-SMA; ACTA2) (Itoga et al., 2003), which facilitate tissue healing. Repeated cycles of inflammation-associated tissue damage followed by tissue remodeling can ultimately lead to fibrosis, a consistent feature observed in endometriosis lesions in women (Matsuzaki and Darcha, 2013; Vigano et al., 2017). Our findings are consistent with a shift in inflammatory status during lesion progression, where, compared to decidualized donor tissue, pro-inflammatory markers predominate at D7, but a more pro-repair phenotype versus decidualized donor tissue is seen at D14.

Despite the shift in inflammatory status, and increased influx of macrophages as predicted in the cellular deconvolution results, our analysis reveals no significant gene expression changes between D7 and D14 lesions. Minor lesion changes in the second week likely involve subsets of cells and could be further impacted by epigenetic regulators including microRNAs (Panir et al., 2018). Transcriptional changes associated with progression of macrophages from a tissue breakdown phenotype to a tissue repair phenotype involves only a small proportion of the total tissue transcriptome, with dynamics that vary according to discrete lesion microenvironments (Panir et al., 2022). Importantly, the current RNA-seq analysis may not be sufficiently sensitive to detect changes in all relevant DEGs. The more sensitive and precise approaches of single-cell RNA-sequencing or spatial transcriptomics would be expected to demonstrate greater changes in different cell types over time. Multiple studies have demonstrated that gene expression changes precede morphological changes (Chapski et al., 2021; Gijbels et al., 1992; Volz et al., 2006), and potentially harvesting lesions at an earlier and/or later timepoint could have allowed detection of more DEGs distinguishing phases of lesion development.

To enable the survival of ectopic endometrial tissues, invasion into the mesothelial layer prior to neovascularization is critical and necessitates modification of the extracellular matrix. Compared to eutopic tissue, ectopic endometrium from women with endometriosis has been shown to constitutively express and secrete several proteins that remodel the extracellular space, such as matrix metalloproteinases (MMPs) (Zhou and Nothnick, 2005). Despite using a model that does not involve mesothelial invasion, *Mmp3* and *Mmp12* are among the top DEGs that were upregulated in the D14 lesions versus decidualized endometrium. In women with endometriosis, polymorphisms in *MMP3* have been linked to an enhanced risk of developing advanced endometriosis and infertility (Cardoso et al., 2019), while polymorphism in *MMP12* is associated with the development of superficial endometriosis (Borghese et al., 2008). This finding suggests that tissue remodeling occurs by 2 weeks post-disease induction in the subcutaneous site, and confirms the importance of MMPs in facilitating attachment to ectopic surfaces and subsequent survival of endometriosis-like lesions. Other studies have indicated that endometriotic lesions in mouse models appear, for the most part, to resolve naturally over time (Dorning et al., 2021). It would be useful to follow the development of lesions to a later timepoint, and to characterize features of lesion architecture that distinguish lesions that successfully establish versus lesions prone to resolve.

Interestingly, the upregulated genes unique to D7 versus decidualized endometrium that are associated with pro-inflammatory macrophage activity include C-X-C motif chemokine 10 (*Cxcl10*), a chemokine involved in the recruitment of immune cells to inflammatory sites (Karin and Razon, 2018) that is also overexpressed in human ectopic endometriosis lesions and associated with neutrophil recruitment (Wang et al., 2023). Interleukin 1 receptor-like 1 (*Il1rl1*) and immunity-related GTPase family M member 1 (*Irgm1*), both upregulated in D7 lesions, are also associated with pro-inflammatory macrophage polarization and activity (Griesenauer and Paczesny, 2017; Nath et al., 2021). Although not implicated in endometriosis thus far, *IRGM* is a known genetic risk factor for several autoimmune diseases (Nath et al., 2021), including Sjogren syndrome and Crohn's disease, raising the possibility of utility as a diagnostic marker for endometriosis. Conversely, several upregulated genes unique to D14 versus decidualized endometrium are associated with pro-repair macrophage activity including *Il4*, a key cytokine that drives pro-repair macrophage polarization (Van Dyken and Locksley, 2013), and chemokine ligand 5 (*Ccl5*), which contributes to the recruitment and activation of these macrophages (Wang et al., 2010). IL-4 present in human ovarian endometriomas can stimulate the proliferation of endometrial stromal cells (OuYang et al., 2008), while the CCL5/CCR5 axis may promote endometriosis progression via T-cell suppression and recruitment of CCR5^+^ myeloid-derived suppressor cells (Zeng et al., 2022). In addition, the observed upregulation of chemokine receptor 7 (*Ccr7*) in D14 lesions may be associated with the activation of adaptive immune responses (Förster et al., 2008). This is consistent with observations that the CCL19/CCR7 axis contributes to the pathogenesis of endometriosis by promoting proliferation and invasion of endometrial stromal cells (Diao et al., 2017).

One of the strengths of our analysis is the identification of several endogenous upstream regulators with potential to cause the observed differential expression of genes in lesions. This information provides clues to the underlying regulatory mechanisms potentially driving pathogenesis. The prominence of interferons (IFNs) in the mouse model [including IFNG and the Interferon alpha and Interferon alpha and beta receptor (Ifnar) groups] mirrors features of human endometriosis, where elevated *IFNA1/2* and *IFNAR2* mRNA are reported in the eutopic endometrium compared to in the endometrium of women without disease (Kao et al., 2003). Elevated JAK1, a key modulator of type I IFN signaling, is observed in ectopic versus eutopic endometrium (Matsuzaki et al., 2006), suggesting that elevated IFN/IFNAR2/JAK1 signaling may be central (Park and Han, 2022). *In vitro* administration of IFN-2B (IFN2) and IFNβ-1A (IFNB1) has been shown to significantly reduce the proliferation and migration of ectopic endometrial stromal cells, with high doses of IFNβ-1A inducing cell cycle arrest and apoptosis (Badawy et al., 2001; Dicitore et al., 2018). Although promising, the therapeutic efficacy of IFN for treatment of endometriosis remains ambiguous. Decreased disease severity, regression of lesions and improved fertility has been reported in women administered IFNα-2B (IFNA2) (Ali et al., 2000), while others noted a higher recurrence rate after IFNα-2B administration (Acién et al., 2002). Evidence of a comparable role for interferon-regulated pathways in the mouse model substantiates its application for investigating molecular mechanisms of dysregulated IFN signaling, and potential for evaluating interventions targeting IFN-mediated effects.

The identification of several additional upstream regulators including microRNAs, cytokines, transcription factors, receptors and growth factors is consistent with the significance of the immune system in modulating disease progression (Fig. S6). Most of the immune regulatory molecules identified are associated with macrophages. Macrophage activation and function is dependent on multiple signals and may fluctuate over the course of disease progression (Cassetta et al., 2011). Although the significance of pro-inflammatory versus pro-healing macrophages in the different stages of endometriosis is clear, activation pathways and detailed phenotypic changes over time have not been delineated, and, reasonably, some of the newly identified upstream regulators could be involved. The sequence of causal pathways linking macrophage phenotype with disease development can only be defined in animal models, in which sequential changes in lesion establishment and immune profiles can be evaluated. In addition, upstream transcriptional regulators and epigenetic modifiers, such as microRNAs and other non-coding RNAs, are known to be dysregulated in endometriosis (Panir et al., 2018). Valuable etiological insight into endometriosis pathophysiology might be provided by investigating which transcriptional regulators modify macrophage polarization during lesion development, for example using donor tissue or recipient mice with null mutations in candidate factors.

A systematic literature search revealed several candidate upstream regulators that have not been previously investigated in the context of endometriosis. Notably, interferon regulatory factor 2-binding protein 2 (IRF2BP2), known for its roles in attenuating macrophage-mediated inflammation and various transcriptional regulations, including cell death, angiogenesis and tumorigenesis, is associated with the activation of Krüppel-like factor 2 (KLF2), an anti-inflammatory transcription factor crucial for vascular integrity and endothelial barrier maintenance (Chen et al., 2015a; Ellegast et al., 2022; Jiang and Shen, 2021; Pastor et al., 2021). In a mouse atherosclerosis model, IRF2BP2-deficient macrophages exhibited pro-inflammatory traits that responded to KLF2 restoration with enhanced anti-inflammatory features (Chen et al., 2015a). We observed upregulation of *Klf2* in D14 lesions versus decidualized endometrium, suggesting that interaction between IRF2BP2 and KLF2 might serve to mitigate excessive inflammation and immune dysregulation. An additional upstream regulator, zinc finger and BTB domain-containing 10 (ZBTB10), affects cell cycle regulation, apoptosis and tumor angiogenesis (Lai et al., 2013). Overexpression of ZBTB10 in human ovarian cancer cell lines led to suppression of FSH-induced angiogenesis by downregulating VEGF, COX2 and survivin [baculoviral inhibitor of apoptosis repeat-containing 5 (BIRC5)] (Lai et al., 2013). Given the established role of ZBTB10 in malignancy and its impact on angiogenesis, defining its involvement in endometriosis could offer crucial insights into disease mechanisms and potentially inform new therapeutic approaches.

Likewise, the non-endogenous upstream regulators warrant examination for candidate therapeutic agents with potential for clinical application. Identification of drugs N-acetyl-L-cysteine, halofuginone and levodopa among the inhibited upstream regulators highlights the potential for investigating repurposing of existing pharmaceuticals for endometriosis treatment. N-acetyl-L-cysteine, for instance, has been investigated for its antioxidant properties and its ability to modulate inflammation, mechanisms relevant to endometriosis pathogenesis (Anastasi et al., 2023). Halofuginone, a compound with anti-inflammatory and anti-fibrotic properties (Wang et al., 2020), might have utility in inhibiting angiogenesis and reducing lesion size. Levodopa, primarily used in the treatment of Parkinson's disease (Tomlinson et al., 2010), exerts its effects through dopamine modulation, which may have implications for pain management and neuroinflammation in endometriosis. Repurposing clinically approved drugs offers a promising avenue for expedited therapeutic development, by leveraging existing safety profiles and pharmacokinetic data.

We acknowledge the limitations inherent in this mouse model. Specifically, although we utilized decidualized endometrium from hormonally cycled animals, the maintenance of recipient mice under relatively constant estradiol levels deviates from the cyclic hormonal fluctuation characteristic of fertile women. This departure from physiological hormonal dynamics may impact the molecular and cellular responses of endometrial tissue, potentially influencing gene expression patterns and the pathophysiological manifestation of endometriosis. Furthermore, the subcutaneous location of the lesions, while facilitating experimental reproducibility and ease of monitoring, may limit aspects of translational relevance specific to the peritoneal cavity. We recognize the importance of considering these limitations in interpreting the results and caution that mouse models should be utilized as one of an armory of experimental approaches to investigate endometriosis.

To summarize the findings, we demonstrate transcriptional changes that occur during development of endometriosis-like lesions in a mouse model of endometriosis. Notwithstanding the limitations of the model, the data presented align with transcriptional changes in human endometriosis, so in our view the model is relevant for investigating aspects of human disease where a shared transcriptional program is evident. In particular, the gene expression changes reflect a central role for the immune system in endometriosis pathophysiology, and significant roles for macrophages as a consistent feature and driving force in lesion establishment and persistence. The study therefore supports the use and validity of this mouse model for investigating the immunology of endometriosis and several related elements of endometriosis pathogenesis, and corresponding potential therapeutic interventions. The provided dataset will be a valuable resource in informing future research questions regarding dysregulated molecular expression and signaling mechanisms contributing to endometriosis development. In particular, the novel upstream regulators we have identified warrant further investigation for their significance in disease etiology and progression.

## MATERIALS AND METHODS

### Animals

All mice used in this study were C57Bl/6Arc (C57Bl/6) females obtained from the Animal Resource Centre (Perth, WA, Australia), kept in group housing and maintained under specific pathogen-free conditions in the Laboratory Animal Services facility at the University of Adelaide, South Australia. Mice were maintained on a 12 h light/12 h dark cycle with sterile breeder chow (10% fat) food and water available *ad libitum*. Sterile filter cages were cleaned and changed weekly, or immediately following operative procedures. All experimental mice were weighed and checked daily to monitor condition and healthy appearance. All animals were used according to the Australian Code of Practice for the Care and Use of Animals for Scientific Purposes (8th edition, 2013), with approval from the Animal Ethics Committee, The University of Adelaide (Ethics identifier: m-2015-040). Genetically Modified Organisms Dealing Authorization was obtained from the Institutional Biosafety Committee, The University of Adelaide (Identifier number: 13354). Prior to commencing experimental procedures, mice were given at least 1 week to recover from transportation and to acclimatize to the facility.

### Mouse model of endometriosis

To establish endometriosis in mice, a modified version of a previously reported menstrual mouse model of endometriosis (Greaves et al., 2014) was used (Fig. 1A). Briefly, mice between 8 and 10 weeks of age were ovariectomized under sterile conditions. Following ovariectomy (D0), mice were randomly allocated into either the donor or recipient group and given a minimum of 7 days to recover prior to commencing the experimental protocol. To induce ‘menses-like’ endometrial tissue, donor mice were given injections of estrogen and implanted with a slow-release progesterone pellet. Decidualization of the endometrium was induced on D15 using 10 μl sesame oil injected trans-cervically into the uterus using a non-surgical embryo transfer device. On D19, donor mice were euthanized 4 h following the withdrawal of progesterone (removal of the pellet). Approximately 40 mg (±2 mg) of decidualized donor endometrial tissue was finely diced, resuspended in 200 µl PBS, and injected subcutaneously into the lower-right quadrant of the ventral abdominal region of recipient mice. In addition, ∼10 mg donor decidualized tissue was either fixed in 4% formalin and processed for histological analysis or snap-frozen in liquid nitrogen for RNA extraction. Endometriosis-like lesions were harvested at one of two timepoints – D7 or D14 after implantation. Photographs of the subcutaneous lesions were taken (Fig. 1B,C), and the lesion was carefully dissected from the site of attachment, weighed and measured to determine lesion width, length and height. Lesions were either fixed in 4% formalin and processed for histological analysis or snap-frozen in liquid nitrogen and subsequently stored at −80°C for RNA extraction.

### Hematoxylin and Eosin staining

Hematoxylin and Eosin (H&E) staining was carried out as per standard protocols. Briefly, slides were stained in Harris Hematoxylin for up to 3 min, followed by a counterstaining in Eosin Y for up to 1 min. Slides were imaged using a Nanozoomer-XR Digital slide scanner (Hamamatsu Photonics). Viewing and analysis of captured images was carried out on the NDP.view2 Viewing software (Hamamatsu Photonics).

### RNA extraction

Total RNA from donor decidualized endometrial tissue, D7 and D14 lesions (four biological replicates at each timepoint; total of 12 individual samples) was extracted from snap-frozen tissue samples using a Qiagen miRNeasy^®^ RNA extraction and purification kit, following the manufacturer's protocol. Contaminating DNA was removed from the sample using commercially available DNase treatment TURBO DNA-free (Life Technologies, Carlsbad, CA, USA) following the manufacturer's instructions. The final concentration of DNase-treated RNA was determined using a Nano-drop Spectrophotometer, and RNA integrity and purity was assessed on an RNA Agilent Bioanalyzer (Agilent Technologies, Santa Clara, CA, USA). All RNA preparations had an RNA integrity number (RIN) of ≥7 and were stored at −80°C until required.

### Library preparation, mRNA sequencing and bioinformatics analysis

Library preparation and mRNA sequencing was performed at the David Gunn Genomics Facility (South Australian Health and Medical Research Institute, Adelaide, SA, Australia). Libraries for a total of 36 samples (four biological samples from each timepoint) were made from ∼1 µg total RNA, quantified by Qubit RNA Assay in a Qubit 2.0 Fluorometer (Life Technologies). Following the manufacturer’s protocol, the mRNA sequencing library was prepared using Illumina's TruSeq RNA Sample Preparation Kit (Illumina, San Diego, CA, USA) for PolyA+ selection. mRNA sequencing of the library preparations was performed on an Illumina Next-Seq 500 to obtain paired-end 75 bp reads at an average depth of 50 million reads per sample. The number of reads obtained per sample is provided in Table S8; PCA for all samples is shown in Fig. S1.

Illumina RNA sequencing data was processed using an in-house RNA-seq workflow. Quality control was carried out using FastQC, followed by quality and adapter trimming using AdapterRemoval2 (Schubert et al., 2016), aligned to the GENCODE mm10 (GRCm38.p4) mouse reference genome using HISAT2 (Kim et al., 2015) and quantified to mm10 annotation using featureCounts (Liao et al., 2013, 2014). Additional sample quality control and normalization were performed using the R Statistical Software Suite, and differential expression analyses were carried out using the R packages limma (Ritchie et al., 2015; Smyth, 2005) and edgeR (Robinson et al., 2010) on a total of 36 biological samples, comprising 12 samples each from C57Bl/6 mice, *miR-155^−/−^* mice and *miR-223^−/−^* mice (refer to Fig. S1 and Table S8). Although all samples were analyzed together to minimize batch effects and technical variations inherent in large-scale transcriptomic studies, for the purposes of this study, only data from the C57Bl/6 mouse strain are presented to ensure sufficient statistical power, reduce potential confounding variables and establish a baseline reference owing to its well-characterized genetic background. Significance was inferred from adjusting raw *P*-values using the Benjamini–Hochberg false discovery rate method (Benjamini and Hochberg, 1995) to yield an FDR, with ≤0.05 being the chosen cut-off. Using the limma::treat function, significance was determined based on a null hypothesis of log_2_FC≥±1. IPA software (2023; Qiagen) was used to identify enriched cellular and molecular functions among DEGs, identify upstream regulators of genes and further classify these genes into functionally related groups. Cellular deconvolution with Euclidean clustering was performed using the mMCP-counter package in R, the murine version of MCP-counter, a tool to estimate the immune and stromal composition of heterogeneous tissue from transcriptomic data (Petitprez et al., 2020). DEGs common to both D7 and D14 versus decidualized endometrium (log_2_FC≥±2, FDR≤0.05) were identified and converted into human orthologous genes (927 upregulated and 526 downregulated) using BioMart. A randomly selected subset of immune-associated genes was chosen using the sample() function in R, resulting in the selection of 54 upregulated genes and 64 downregulated genes for analysis (Fig. S2). The expression of these orthologous genes was evaluated in publicly available human datasets, utilizing the EndometDB database, an interactive web-based platform that integrates expression data from 115 patients and 53 controls (Gabriel et al., 2020).

### Statistical analysis

Additional statistical analyses for morphometric measurements were performed using GraphPad Prism version 10. As the D'Agostino and Pearson normality test (Pearson et al., 1977) revealed a non-parametric distribution, the Mann–Whitney *U*-test (Mann and Whitney, 1947) was used to determine statistical significance. Data are presented as median (interquartile range), and significance was inferred at *P*≤0.05.

## Supplementary Material

10.1242/dmm.050566_sup1 Supplementary information

Table S1. Differentially Expressed Genes (DEGs) identified in D7 endometriosis-like lesions *vs* decidualized endometrium from C57Bl/6 mice.

Table S2. Differentially Expressed Genes (DEGs) identified in D14 endometriosis-like lesions *vs* decidualized endometrium from C57Bl/6 mice.

Table S3. Differentially Expressed Genes (DEGs) identified in D14 *vs* D7 endometriosis-likelesions from C57Bl/6 mice.

Table S4. Ingenuity Canonical Pathways identified in D7 endometriosis-like lesions *vs* decidualized endometrium from C57Bl/6 mice.

Table S5. Ingenuity Canonical Pathways identified in D14 endometriosis-like lesions *vs* decidualized endometrium from C57Bl/6 mice.

Table S6. Upstream regulators identified in D7 endometriosis-like lesions *vs* decidualized endometrium from C57Bl/6 mice.

Table S7. Upstream regulators identified in D14 endometriosis-like lesions *vs* decidualized endometrium from C57Bl/6 mice.

Table S8. Number of reads generated per RNA sample.
