# Supplementary material for: RNA sequencing reveals molecular mechanisms of endometriosis lesion development in mice
Source: Dis Model Mech. 2024 Oct 23;17(10):dmm050566. doi: 10.1242/dmm.050566 (PMC11524442; doi:10.1242/dmm.050566)
Supplement: Supplementary information [file dmm-17-050566-s1.pdf]

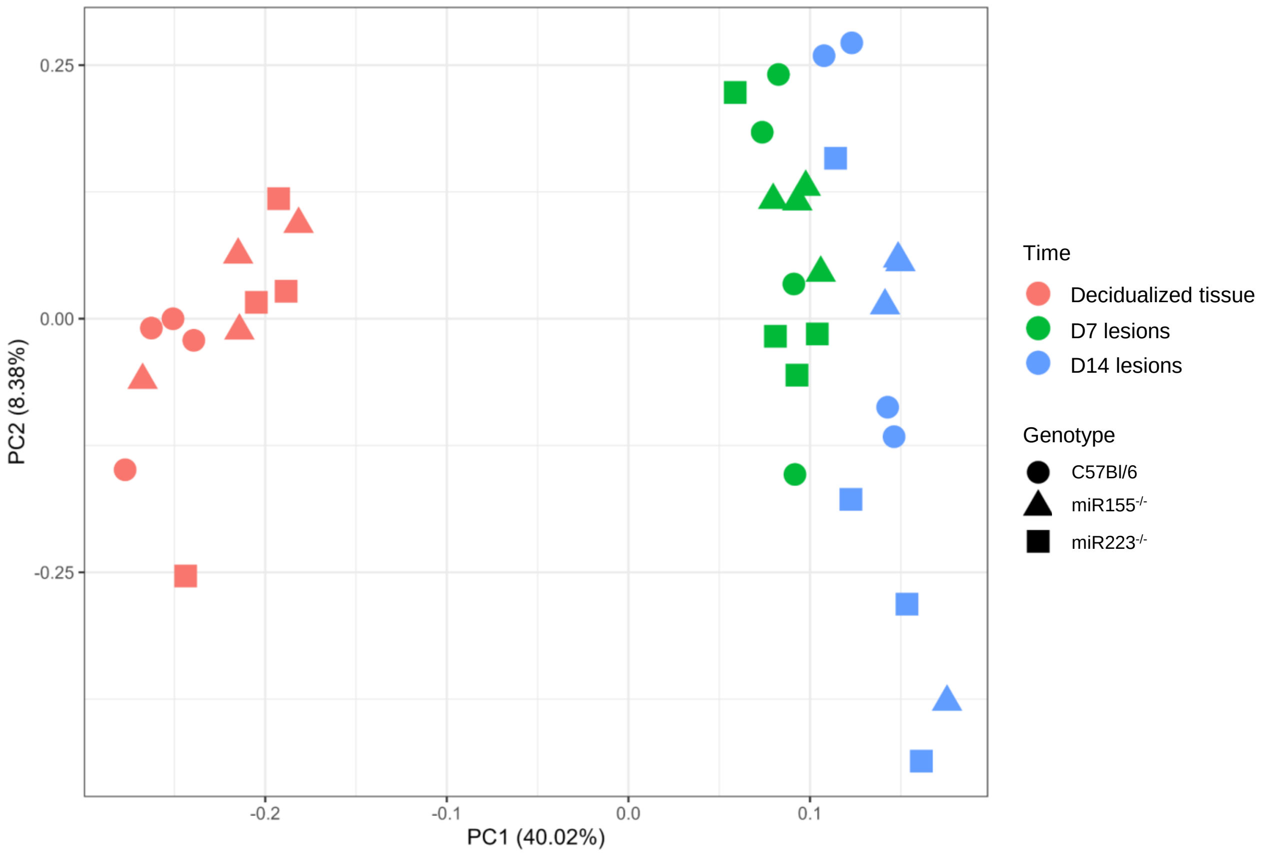

Fig. S1. Principal component analysis plot for all 36 samples used in the analysis. A principal component analysis (PCA) plot was constructed to further explore the relationships amongst the 36 different samples in the dataset, and provides additional evidence of the clear separation between decidualized endometrial samples and lesion samples, reinforcing the robustness of the observed distinctions.

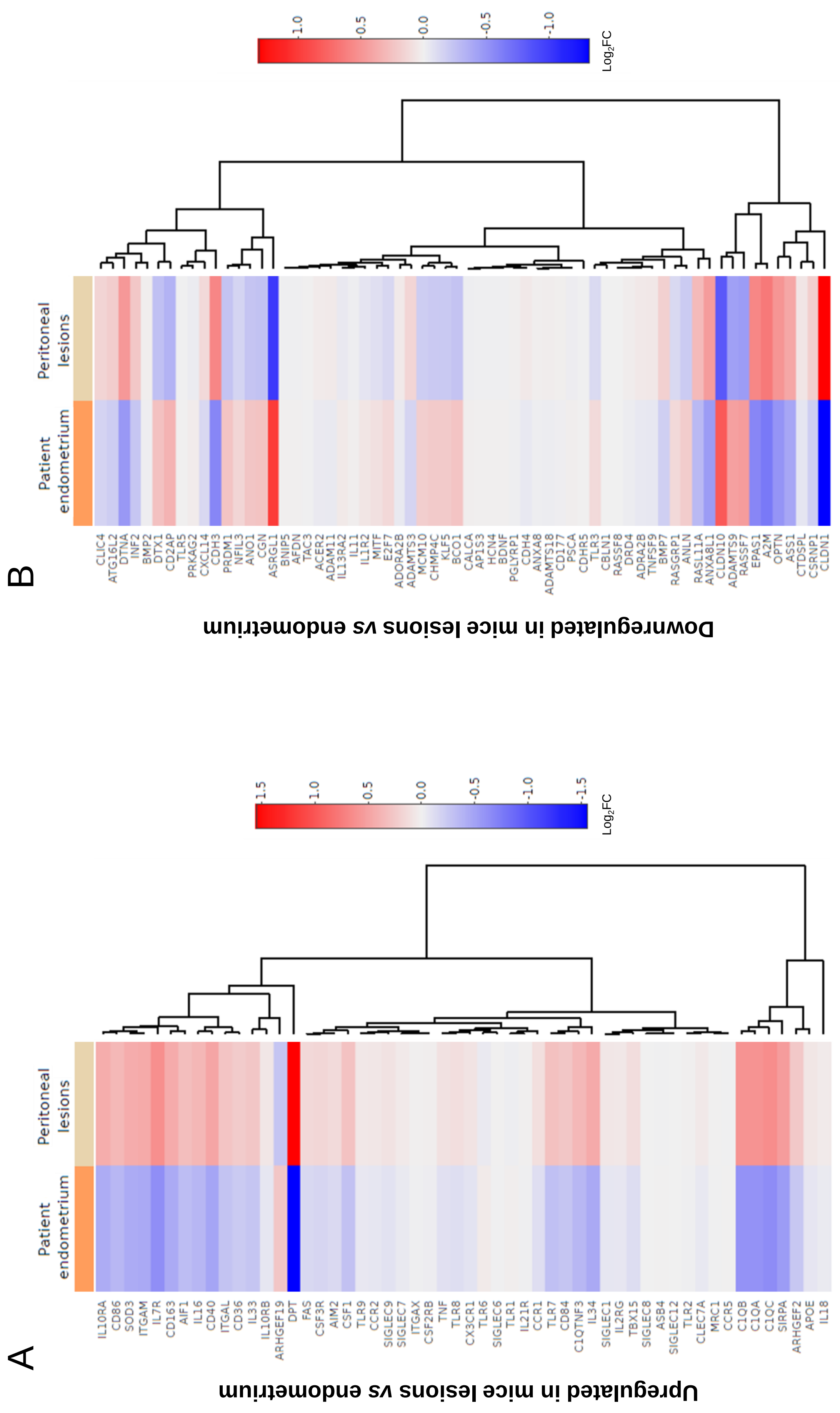

Disease Models & Mechanisms • Supplementary information

Fig. S2. Expression of human orthologues corresponding to mouse genes from our dataset. A selection of DEGs common to both Day 7 (D7) and Day 14 (D14) versus endometrium (log2FC ≥ ± 2, FDR ≤ 0.05) were identified and converted into human orthologous genes using BioMart. The expression of orthologous genes was evaluated in publicly available human datasets comparing patient endometrium and peritoneal lesions, utilizing the EndometDB database (https://endometdb.utu.fi/) and presents as **(A)** Heatmap depicting the expression levels of human orthologues corresponding to upregulated mouse genes, and **(B)** Heatmap depicting the expression levels of human orthologues corresponding to downregulated mouse genes.

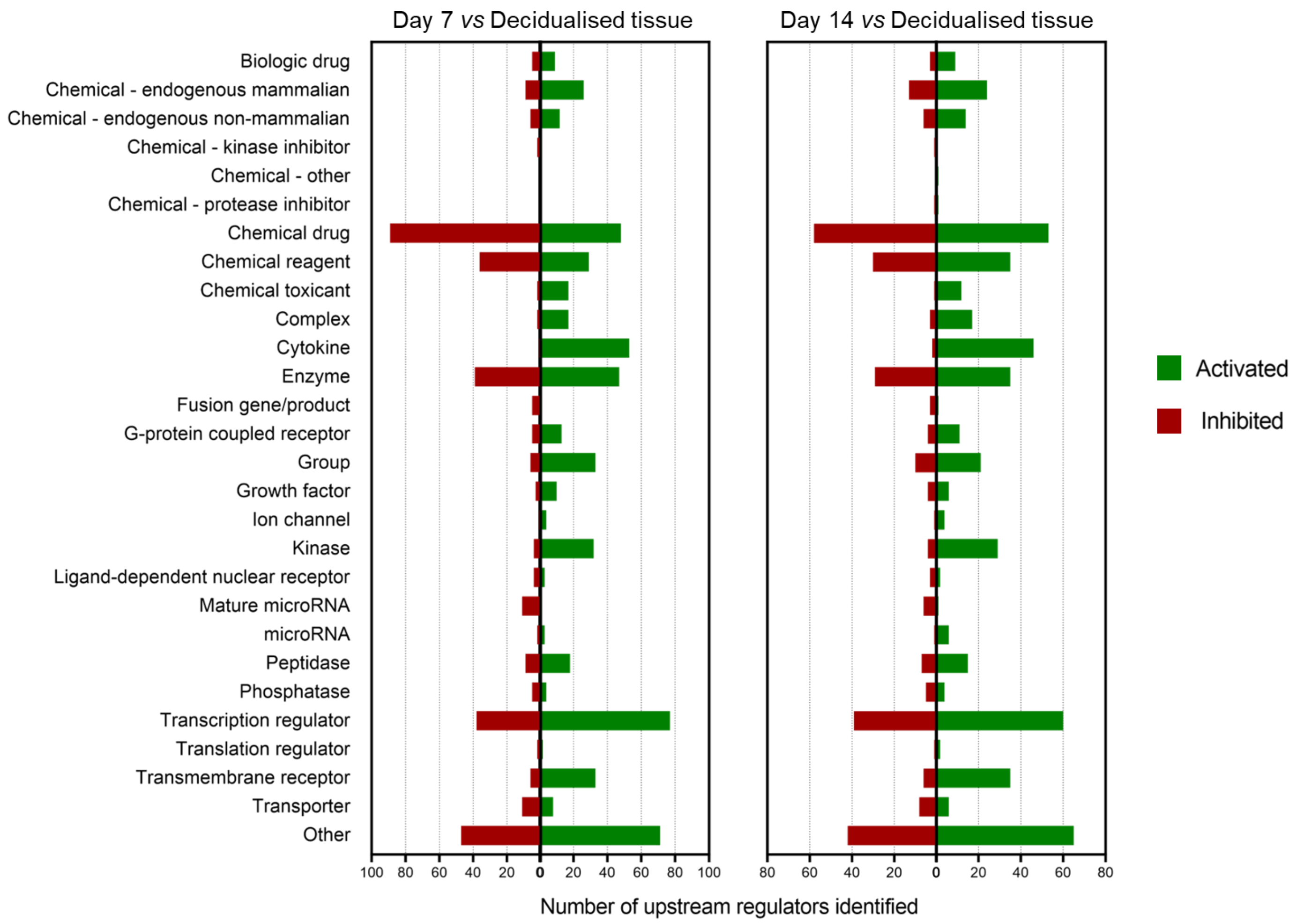

Fig. S3. Classification of molecules predicted to be upstream of genes regulated in Day 7 and Day 14 endometriosis-like lesions vs decidualized endometrium identified by IPA. Predicted upstream regulators were grouped into 28 molecular categories. All upstream regulators have a *Z*- activation score of ≥ +2 or ≤ -2, and corresponding *p*-value of ≤ 0.05.

Disease Models & Mechanisms • Supplementary information

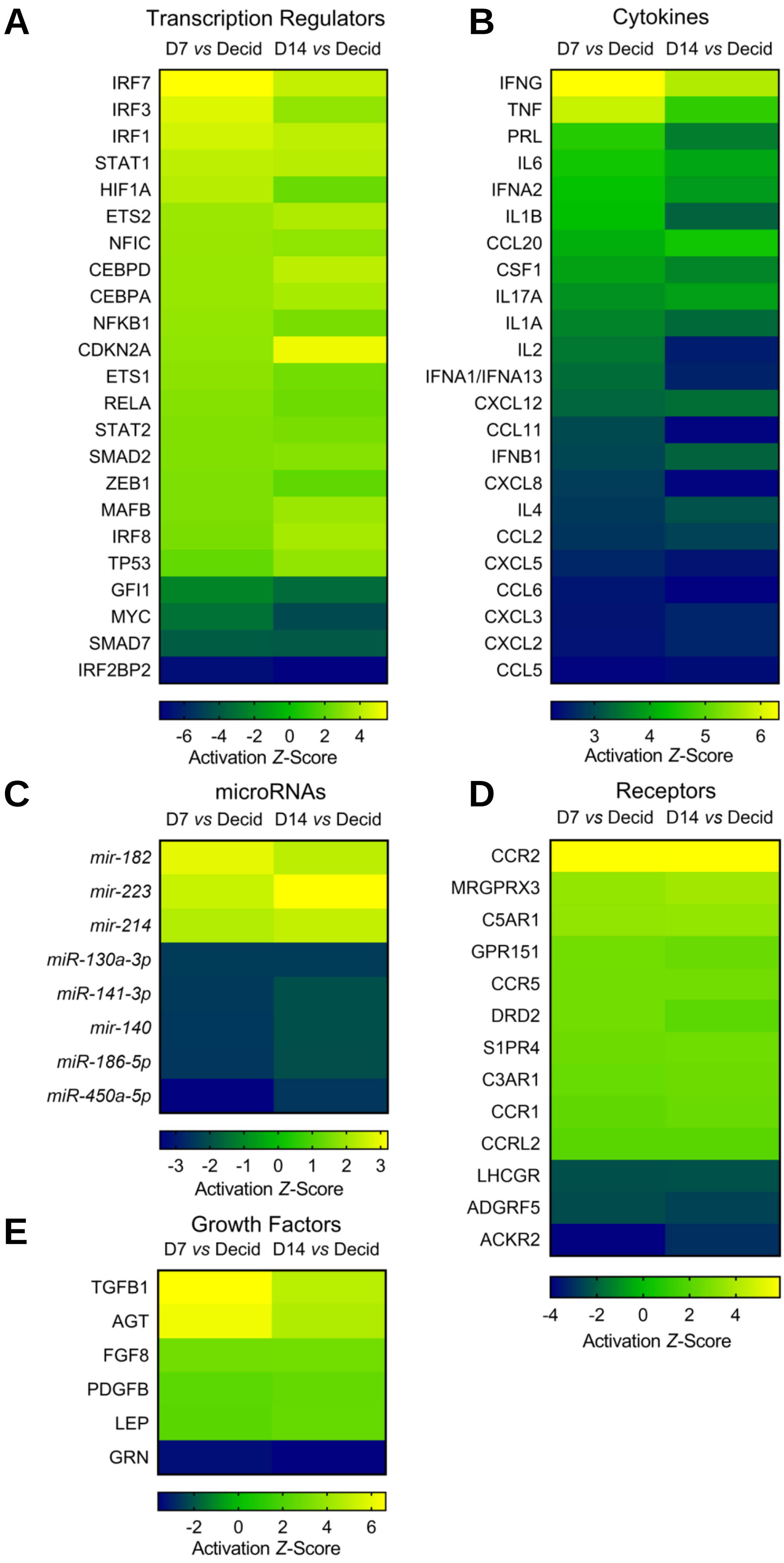
A
Transcription Regulators
D7 *vs* Decid
D14 *vs* Decid
IRF7
IRF3
IRF1
STAT1
HIF1A
ETS2
NFIC
CEBPD
CEBPA
NFKB1
CDKN2A
ETS1
RELA
STAT2
SMAD2
ZEB1
MAFB
IRF8
TP53
GFI1
MYC
SMAD7
IRF2BP2
-6 -4 -2 0 2 4
Activation *Z*-Score
B
Cytokines
D7 *vs* Decid
D14 *vs* Decid
IFNG
TNF
PRL
IL6
IFNA2
IL1B
CCL20
CSF1
IL17A
IL1A
IL2
IFNA1/IFNA13
CXCL12
CCL11
IFNB1
CXCL8
IL4
CCL2
CXCL5
CCL6
CXCL3
CXCL2
CCL5
3 4 5 6
Activation *Z*-Score
C
microRNAs
D7 *vs* Decid
D14 *vs* Decid
mir-182
mir-223
mir-214
miR-130a-3p
miR-141-3p
mir-140
miR-186-5p
miR-450a-5p
-3 -2 -1 0 1 2 3
Activation *Z*-Score
D
Receptors
D7 *vs* Decid
D14 *vs* Decid
CCR2
MRGPRX3
C5AR1
GPR151
CCR5
DRD2
S1PR4
C3AR1
CCR1
CCRL2
LHCGR
ADGRF5
ACKR2
-4 -2 0 2 4
Activation *Z*-Score
E
Growth Factors
D7 *vs* Decid
D14 *vs* Decid
TGFB1
AGT
FGF8
PDGFB
LEP
GRN
-2 0 2 4 6
Activation *Z*-Score

Fig. S4. Predicted upstream regulators of genes identified in D7 and D14 endometriosis-like lesions compared to decidualized endometrium. Upstream regulators of pathways were identified by IPA [*Z*- score of ≥ +2 or ≤ -2] and heatmaps were generated to show comparisons between Day 7 lesions *vs* Decidualized endometrium (Decid) and Day 14 lesions *vs* Decidualized endometrium (Decid) for the categories of **(A)** transcription regulators, **(B)** cytokines, **(C)** microRNAs **(D)** receptors, and **(E)** growth factors. Note: This list is not exhaustive; complete list of upstream regulators are available in the Supplementary Material (Table S6 and S7).

Disease Models & Mechanisms • Supplementary information

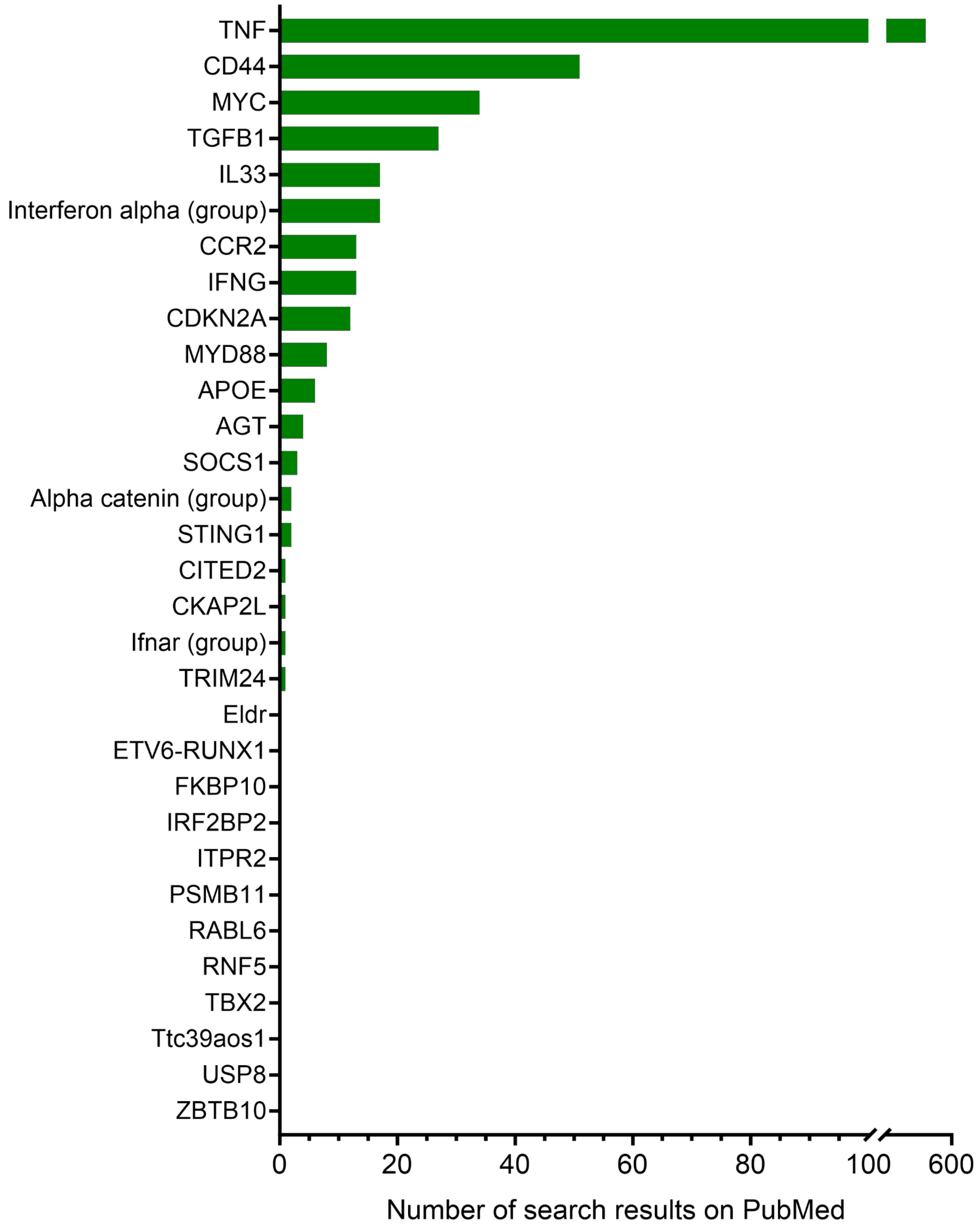
TNF
CD44
MYC
TGFB1
IL33
Interferon alpha (group)
CCR2
IFNG
CDKN2A
MYD88
APOE
AGT
SOCS1
Alpha catenin (group)
STING1
CITED2
CKAP2L
Ifnar (group)
TRIM24
Eldr
ETV6-RUNX1
FKBP10
IRF2BP2
ITPR2
PSMB11
RABL6
RNF5
TBX2
Ttc39aos1
USP8
ZBTB10
0
20
40
60
80
100
600
Number of search results on PubMed

Fig. S5. Number of published studies in the scientific literature reporting top predicted endogenous upstream regulators in comparisons between endometriosis-like lesions and decidualized endometrium. Data depict the number of search results returned via the PubMed Search Engine for individual top 10 activated and inhibited endogenous upstream regulators of endometriosis (see Figure 6A in main paper for details). PubMed searches were performed using the search terms ‘molecule symbol’ AND ‘endometriosis’.

Disease Models & Mechanisms • Supplementary information

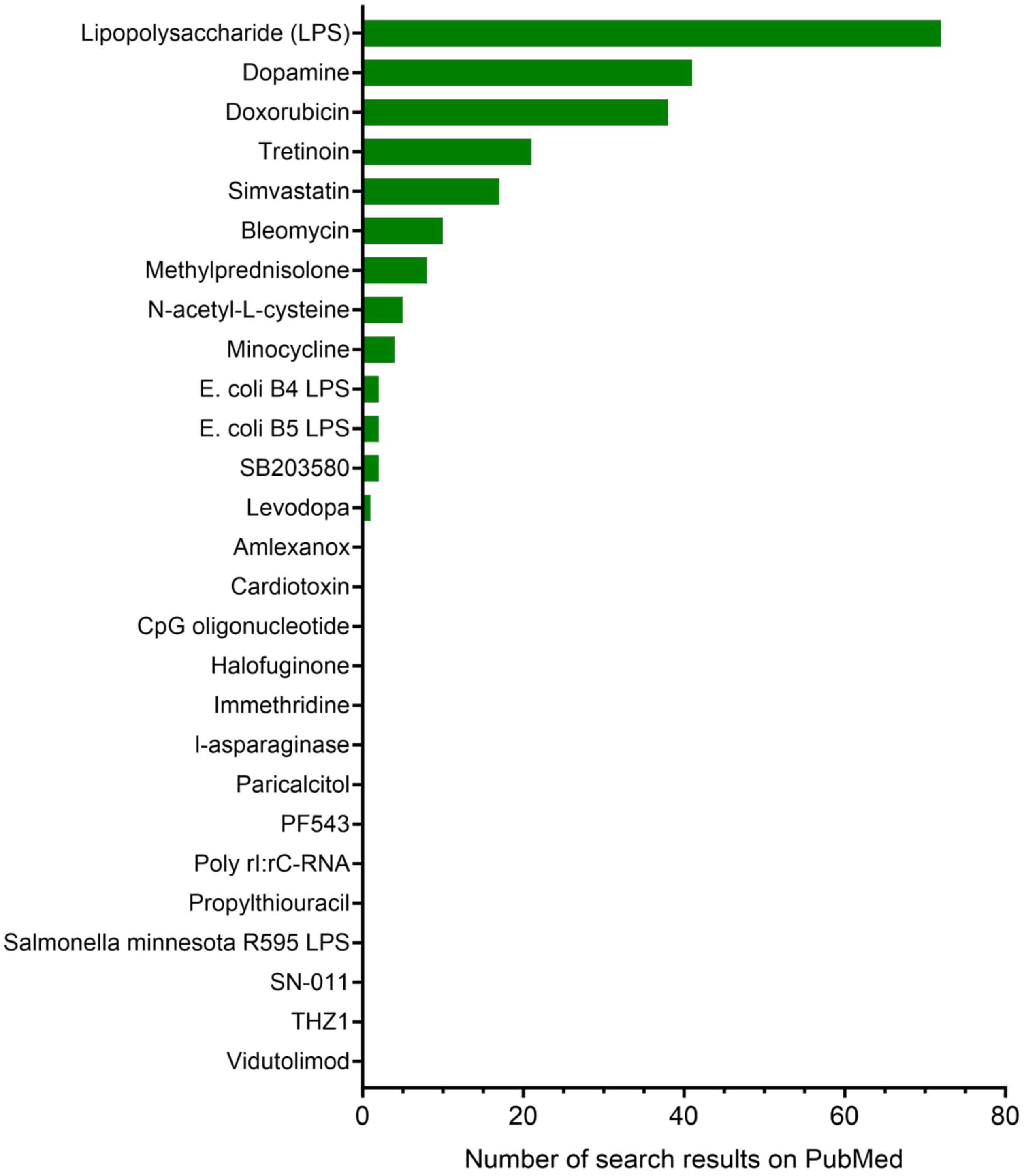

Fig. S6. Number of published studies in the scientific literature reporting top predicted non-endogenous upstream regulators in comparisons between endometriosis-like lesions and decidualized endometrium. Data depict the number of search results returned via the PubMed Search Engine for individual top 10 activated and inhibited non-endogenous upstream regulators of endometriosis (see Figure 6B in main paper for details). PubMed searches were performed using the search terms 'molecule symbol' AND 'endometriosis'.

Disease Models & Mechanisms • Supplementary information

Table S1. Differentially Expressed Genes (DEGs) identified in D7 endometriosis-like lesions *vs* decidualized endometrium from C57Bl/6 mice.

Available for download at https://journals.biologists.com/dmm/article-lookup/doi/10.1242/dmm.050566#supplementary-data

Table S2. Differentially Expressed Genes (DEGs) identified in D14 endometriosis-like lesions *vs* decidualized endometrium from C57Bl/6 mice.

Available for download at https://journals.biologists.com/dmm/article-lookup/doi/10.1242/dmm.050566#supplementary-data

Table S3. Differentially Expressed Genes (DEGs) identified in D14 *vs* D7 endometriosis-like lesions from C57Bl/6 mice.

Available for download at https://journals.biologists.com/dmm/article-lookup/doi/10.1242/dmm.050566#supplementary-data

Table S4. Ingenuity Canonical Pathways identified in D7 endometriosis-like lesions *vs* decidualized endometrium from C57Bl/6 mice.

Available for download at https://journals.biologists.com/dmm/article-lookup/doi/10.1242/dmm.050566#supplementary-data

Table S5. Ingenuity Canonical Pathways identified in D14 endometriosis-like lesions *vs* decidualized endometrium from C57Bl/6 mice.

Available for download at https://journals.biologists.com/dmm/article-lookup/doi/10.1242/dmm.050566#supplementary-data

Table S6. Upstream regulators identified in D7 endometriosis-like lesions *vs* decidualized endometrium from C57Bl/6 mice.

Available for download at
https://journals.biologists.com/dmm/article-lookup/doi/10.1242/dmm.050566#supplementary-data

Table S7. Upstream regulators identified in D14 endometriosis-like lesions *vs* decidualized endometrium from C57Bl/6 mice.

Available for download at
https://journals.biologists.com/dmm/article-lookup/doi/10.1242/dmm.050566#supplementary-data

Table S8. Number of reads generated per RNA sample.

Available for download at
https://journals.biologists.com/dmm/article-lookup/doi/10.1242/dmm.050566#supplementary-data
